# CRISPR based editing of SIV proviral DNA in ART treated non-human primates

**DOI:** 10.1038/s41467-020-19821-7

**Published:** 2020-11-27

**Authors:** Pietro Mancuso, Chen Chen, Rafal Kaminski, Jennifer Gordon, Shuren Liao, Jake A. Robinson, Mandy D. Smith, Hong Liu, Ilker K. Sariyer, Rahsan Sariyer, Tiffany A. Peterson, Martina Donadoni, Jaclyn B. Williams, Summer Siddiqui, Bruce A. Bunnell, Binhua Ling, Andrew G. MacLean, Tricia H. Burdo, Kamel Khalili

**Affiliations:** 1 Department of Neuroscience, Center for Neurovirology, Lewis Katz School of Medicine at Temple University, 3500N. Broad Street, 7th Floor, Philadelphia, PA 19140 USA; 2 Division of Comparative Pathology, Tulane National Primate Research Center, Covington, LA 70433 USA; 3 Tulane Brain Institute, Tulane University, New Orleans, LA 70118 USA; 4 Department of Pharmacology, Tulane University School of Medicine, New Orleans, LA 70112 USA; 5 Department of Microbiology, Immunology and Genetics, University of North Texas Health Science Center, Fort Worth, TX 76107 USA; 6 Department of Microbiology & Immunology, Tulane University School of Medicine, New Orleans, LA 70112 USA; 7 Texas Biomedical Research Institute, San Antonio, TX 78227 USA

**Keywords:** Gene therapy, Drug delivery, HIV infections, Experimental models of disease

## Abstract

Elimination of HIV DNA from infected individuals remains a challenge in medicine. Here, we demonstrate that intravenous inoculation of SIV-infected macaques, a well-accepted non-human primate model of HIV infection, with adeno-associated virus 9 (AAV9)-CRISPR/Cas9 gene editing construct designed for eliminating proviral SIV DNA, leads to broad distribution of editing molecules and precise cleavage and removal of fragments of the integrated proviral DNA from the genome of infected blood cells and tissues known to be viral reservoirs including lymph nodes, spleen, bone marrow, and brain among others. Accordingly, AAV9-CRISPR treatment results in a reduction in the percent of proviral DNA in blood and tissues. These proof-of-concept observations offer a promising step toward the elimination of HIV reservoirs in the clinic.

## Abstract (web summary)

Removal of integrated HIV DNA remains a roadblock for HIV cure. Here, Mancuso *et al*. show that intravenous administration of an adeno-associated virus-based CRISPR/Cas9 gene editing construct to SIV-infected macaques results in excision of integrated proviral DNA from infected blood cells and tissues known to be viral reservoirs.

## Introduction

Advances in antiretroviral therapy (ART) have led to great efficacy in limiting replication of resident HIV-1, reducing plasma viral load and improving immune system function^1,2^. However, ART cannot permanently eliminate the virus and cure the illness caused by viral infection and people with HIV (PWH) experience viral load rebound within weeks of ART interruption^3^. Moreover, PWH on ART remain vulnerable to a broad range of comorbidities, in part, associated with treatment regimens and the presence of copies of the proviral DNA that are integrated into the host chromosomes and remain able to express toxic viral proteins^4^. These observations prompted us to employ a genetic strategy involving gene editing to compromise the integrity of the proviral DNA and as a result inactivate its expression. To achieve this goal during the past 5 years, we have adapted CRISPR methods for targeting and editing various regions within the HIV-1 genome^5^ and here, the SIV genome, and evaluated their ability to alter viral gene expression and replication in cultured cells and animal models^6,7^. For in vivo delivery, we created a platform using an adeno-associated virus (AAV)-mediated gene delivery vector that is widely distributed to a broad range of tissues and allows for the simultaneous expression of the Cas9 endonuclease and multiple guide RNAs (gRNAs) that specifically recognize Gag and LTR regions of the viral genome^8^. The use of an all-in-one gRNAs-Cas9 vector facilitated uniform delivery and expression of the multiple components required for the editing and efficient removal of large segments of the integrated viral DNA spanning between the cleavage sites from the host genome, which mitigates the chance for the emergence of replication competent virus^9,10^.

## Results

### Construction of SIV-specific CRISPR-based editing constructs in cell culture

**Fig. 1:**
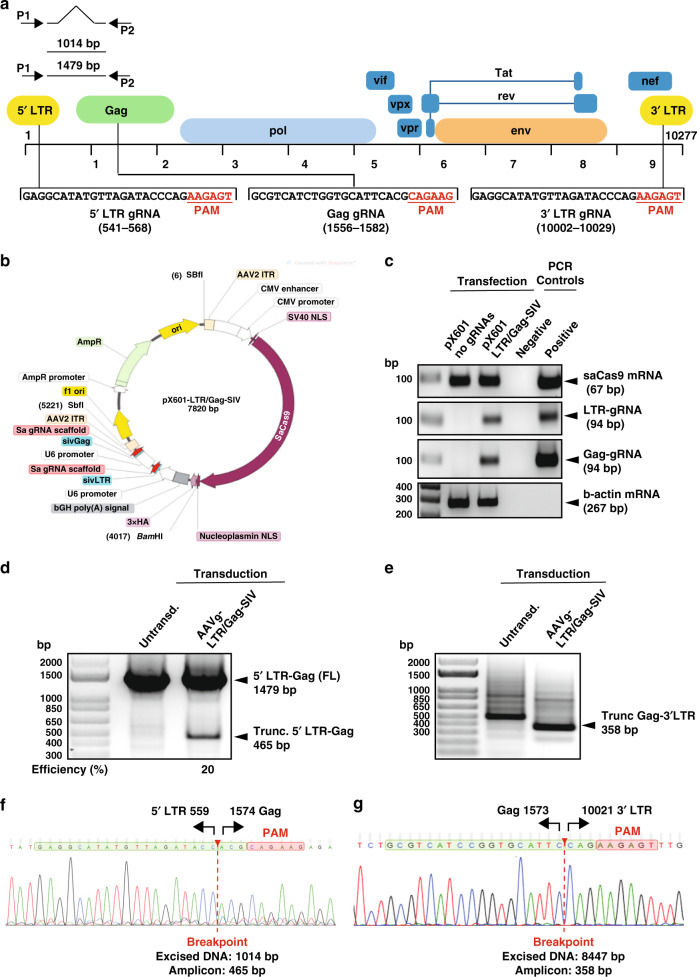
Construction, map and confirmation of the CRISPR-Cas9 construct targeting SIV proviral DNA in vitro. **a** Schematic presentation of the full-length SIVmac239 genome (GenBank: M33262.1) with positions of targeted sites, predicted cleavage events and gRNAs sequences. **b** Map of AAV pX601-SaCas9-2xgRNA plasmid. **c** Confirmation of the expression of the plasmid SaCas9 and gRNAs. RT-PCR analysis of SaCas9 and gRNA expression in HEK293T cells transfected with the plasmid. **d** Gel electrophoresis analysis of PCR reaction for detection of SIV DNA after the treatment of cells with AAV9 delivering CRISPR-Cas9. Results from the PCR analysis showed a clear band of 465 bp in size in the cells that received the CRISPR-Cas9 by infection with AAV9 expressing CRISPR-Cas9. The percent excision efficiency (Efficiency (%)) in vitro shown under the PCR was calculated by quantification of the excised band (trunc.) divided by the  sum of the full-length (FL) plus the excised bands  times 100%. **e** Results from the PCR analysis showed a clear band of 358 bp in size in the cells that received the CRISPR-Cas9 by infection with AAV9 expressing CRISPR-Cas9. **f** Results from the sequencing of the 465 bp amplicon showed the breakpoint of the viral DNA, where the truncated 5′LTR is joined to the residual Gag gene after the removal of the 1014 bp DNA. **g** Results from the sequencing of the 358 bp amplicon showed the breakpoint of the viral DNA, where the truncated gag is joined to the residual 3′LTR after the removal of the 8447 bp DNA. Full sequencing data are available in the source file data provided with this paper. Source data are provided as a Source Data file.

To create the SIV-specific editing molecule, first we employed a bioinformatics tool to identify a pair of nucleotide sequences, similar to those used for HIV-1 editing (https://www.benchling.com), to develop a pair of gRNAs with the highest predicted on-target and lowest scores for off-target cleavage within the 5′ LTR and gag gene of the SIVmac239 genome. Efficient guiding of Cas9 to the designated sites for cleavage can lead to excision of the intervening segment of viral DNA from the infected host genome. Successful editing of the integrated viral DNA at the 5′ and 3′ LTRs may result in the removal of the full-length viral genome or generation of transcription/replication defective proviral DNA that lacks sequences between the 5′ LTR to gag or gag to 3′ LTR sites (Fig. 1a). To create the therapeutic plasmid, the protospacer regions for both gRNAs were cloned into a single AAV9-_Sa_Cas9 vector and after verification of their expression in HEK293T (Fig. 1b, c), cells were infected with EcoSIVmac239e-Luciferase/VSV-g reporter virus for assessing its gene editing capacity. PCR amplification of targeted 5′ LTR-gag and gag-3′ LTR regions of the SIV genome in the treated cells revealed the presence of 465 and 358 bp products, respectively (Fig. 1d, e). Results from Sanger sequencing verified the removal of the intervening DNA fragments of 1014 bp (5′ LTR-gag) and 8447 bp (gag-3′ LTR) between the cleavage sites (Fig. 1f, g).

### Ex vivo editing of SIV DNA in  peripheral blood mononuclear cells (PBMCs) of infected animals

**Fig. 2:**
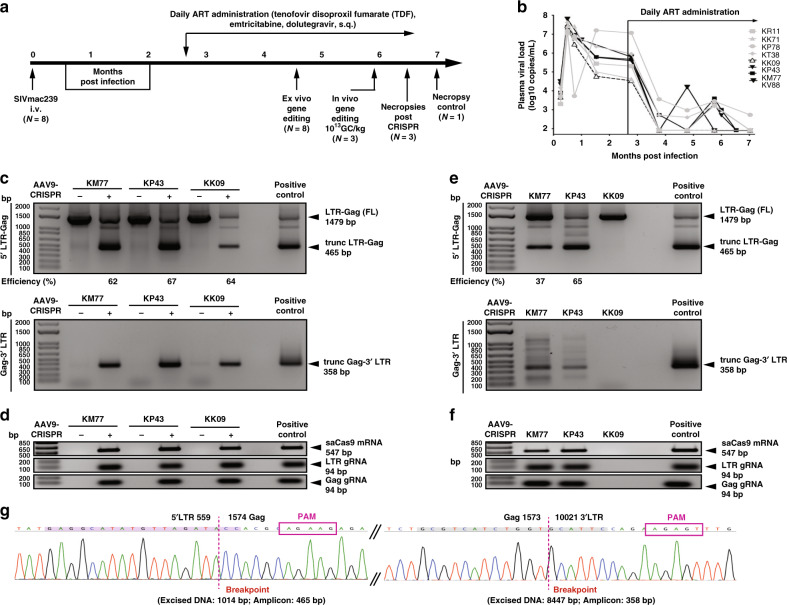
Excision of SIVmac239 DNA by CRISPR-Cas9 in the blood of SIV-infected rhesus macaque monkeys. **a** Chinese rhesus macaques were i.v. inoculated with SIVmac239 and given a daily ART administration of tenofovir disoproxil fumarate, emtricitabine and dolutegravir, s.q. starting at 78 days post infection. Three animals (KM77, KP43 and KV88) were given 10^13^ GC/kg, i.v. and 3 weeks later underwent necropsy. One animal served as the no CRISPR control (KK09). **b** Plasma viral loads of the SIV-infected ART-treated rhesus macaques. The threshold of the assay was 83 copies/ml. The animals in solid black symbols and lines received AAV9/CRISPR/Cas9. The open black triangle with a dotted line (KK09) was sacrificed as no AAV9/CRISPR control. The gray symbols and lines were animals that were only used in the ex vivo screening. All viral loads were done in duplicate per time point. **c** AAV9-mediated delivery of CRISPR/Cas9 to ex vivo PBMCs from SIVmac239-infected animals was able to excise SIV viral DNA. Truncated 5′LTR-gag (465 bp) and gag-3′LTR (358 bp) amplicons were detected in lanes with AAV-9 CRISPR/Cas9 (+) but not in the untransformed (−). The percent excision efficiency ex vivo (Efficiency (%)) shown under the PCR was calculated by quantification of the excised band (Trunc.) divided by the  sum of the  full-length band (FL)  plus the excised bands times 100% (see Supplementary Fig. S1 for all animals). **d** Expression was verified by the presence of SaCas9 mRNA (547 bp), LTR and Gag gRNA scaffolds (94 bp). **e** Similarly, in vivo excision was confirmed in the blood of KM77 and KP43 by the PCR amplification and detection of the trunc 5′LTR to gag (465 bp) and the gag-3′LTR (358 bp) (see Supplementary Fig. S3 for KV88). The percent excision efficiency (Efficiency (%)) in vivo shown under the PCR was calculated as above. KK09 did not receive CRISPR so no efficiency was calculated. **f** Expression was verified by the presence of SaCas9 mRNA (547 bp), LTR and Gag gRNA scaffolds (94 bp). **g** Representative Sanger sequence tracings of 5′LTR-Gag (left) and Gag-3′LTR (right) CRISPR-Cas9 induced truncated SIV-specific amplicons. Target sites are highlighted in green, PAMs motifs in red, the double cleaved/end-joined site is shown as a breaking point in red. Full sequencing data are available in the source file data provided with this paper. Source data are provided as a Source Data file.

Next, we investigated the ability of our CRISPR molecule to edit SIVmac239 DNA in SIV-infected rhesus macaques (Fig. 2a). All animals were pre-screened for pre-existing neutralizing antibodies to AAV9 and were negative. Animals were infected with SIVmac239 and given an ART regimen shown to suppress viral load (2.7 months after infection)^11^ (Fig. 2b). Ex vivo gene editing was performed in PBMCs (*n* = 8) from SIV-infected ART-treated animals at 5 months post infection by transduction with AAV9-_Sa_Cas9:2xgRNA (MOI 2.5 × 10^5^ GC) and PCR amplification to assess the potency and precision of viral DNA elimination (Fig. 2c, d and Supplementary Fig. S1a−e). In PBMCs from all SIV-infected animals, ex vivo excision of viral DNA was confirmed by the detection of single nested PCR products of distinct DNA fragments of 465 and 358 bp resulting from the removal of intervening DNA sequences between 5′LTR to Gag and Gag to 3′LTR, respectively (Fig. 2c and Supplementary Fig. S1a, b). Ex vivo excision efficiency of the 5′LTR-Gag was between 61 and 100%. Expression of Cas9 and gRNAs was also confirmed by RT-PCR (Fig. 2d and Supplementary Fig. S1c).

### In vivo editing of proviral DNA results in the excision of specific fragments of SIV proviral DNA from the host genome in blood and various tissues

Three randomly selected animals (KM77, KP43 and KV88) were then given an in vivo  intravenous (i.v.) infusion of AAV9/CRISPR-Cas9 (10^13^ GC/kg) and one that was manufactured by viral vector core at the University of North Carolina (Fig. 2a). After 3 weeks, blood and tissues were harvested at necropsy. One animal KK09 was necropsied as a no CRISPR control. Viral load (Fig. 2b), lymphocytes, white blood cells, biochemical parameters related to liver and kidney functions, weight and CD4 and CD8 counts were monitored before and after the ART treatments (Supplementary Fig. S2a, b). In vivo, both 5′LTR to Gag (465 bp amplicon) and Gag to 3’ LTR (358 bp amplicon) excision were confirmed in the blood of animals that received AAV9/CRISPR-Cas9 infusion (Fig. 2e and Supplementary Fig. S3a), but not the control animal (KK09), which was SIV infected, ART treated but without AAV9. The in vivo excision efficiency of the 5′LTR-Gag in blood was 37% (KM77), 65% (KP43) (Fig. 2e) and 92% (KV88) (Supplementary Fig. S3a). Expression of Cas9 and gRNAs in vivo was also confirmed by RT-PCR (Fig. 2f and Supplementary Fig. S3b). All amplicons ex vivo (Supplementary Fig. S1d, e) and in vivo were confirmed by Sanger sequencing (Fig. 2g and Supplementary Fig. S3c).

### Biodistribution of CRISPR in the infected animals

**Fig. 3:**
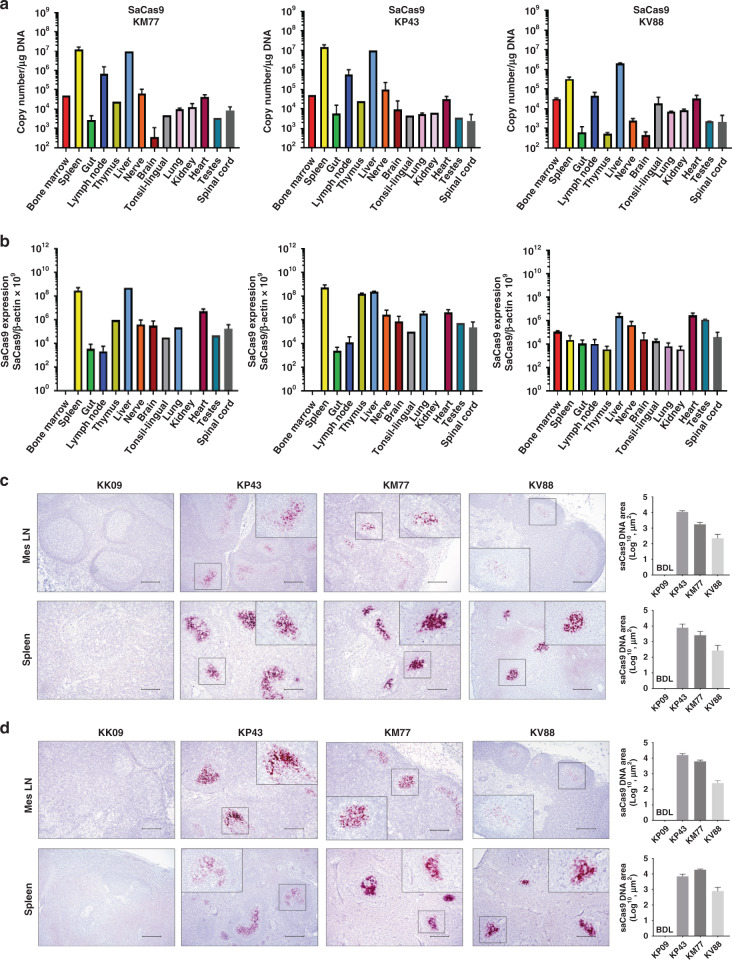
Biodistribution of CRISPR-Cas9 vector in tissues. **a** ddPCR analysis of Cas9 transgene DNA levels in genomic DNA extracted from various tissues of AAV9-CRISPR-SIV-treated animals (KM77, KP43 and KV88). **b** Detection of Cas9 RNA as a ratio to β-actin in various tissues of three animals i.v. inoculated with AAV9-CRISPR. Fifty nanograms of cDNA was analyzed in each reaction well for measuring the expression of SaCas9. β-Actin as reference gene for copy number reference reactions. Data in panels (**a**) and (**b**) are shown as mean ± SEM. Number of samples for each tissue is as follows: *n* = 1 for liver, tonsil and bone marrow; *n* = 12 for gut; *n* = 8 for lymph node; *n* = 6 for spinal cord; *n* = 9 for brain; *n* = 3 for spleen; *n* = 4 for heart; *n* = 2 for nerve, kidney, and lung. All samples were run in duplicate. **c** Using DNAScope technology, Cas9 DNA was detected in the mesenteric lymph nodes and spleens of SIV-infected ART-treated rhesus macaques with in vivo AAV-9-CRISPR administration (KP43, KM77 and KV88), but not in the animal without in vivo AAV-9-CRISPR (KK09). Cas9 DNA was quantified using Keyence BZ-X700 Microscope and accompanying Batch Analysis Software to determine the average area of positive signal from 30 nonoverlapping ×10 images. Data are presented as mean values of the 30 counts ±SEM. Representative images are ×10 with inserts at ×40 magnification. BDL below detection limit. **d** Cas9 RNA in tissues from rhesus macaques treated with AAV9-CRISPR in vivo. Using RNAScope technology, Cas9 RNA was detected in the mesenteric lymph nodes (Mes LN) and spleens of SIV-infected ART-treated rhesus macaques with in vivo AAV-9-CRISPR administration (KP43, KM77 and KV88), but not in the animal without in vivo AAV-9-CRISPR (KK09). Cas9 RNA was quantified using Keyence BZ-X700 Microscope and accompanying Batch Analysis Software to determine the average area of positive signal from 30 nonoverlapping ×10 images. Data are presented as mean values of the 30 counts ±SEM. Representative images are ×10 with inserts at ×40 magnification. BDL below detection limit. Scale bar = 200 μm Source data are provided as a Source Data file.

Representative tissues from the two necropsied animals were used for biodistribution of Cas9 DNA and RNA (Fig. 3). Results from ddPCR showed Cas9 DNA (Fig. 3a and Supplementary Fig. S4) and RNA (Fig. 3b and Supplementary Fig. S4) in a broad range of tissues, including bone marrow, spinal cord, tonsil, brain, spleen, gut, liver, lymph node, thymus and heart. In corroboration with the ddPCR observations, results from the DNAScope (Fig. 3c) and RNAScope (Fig. 3d) illuminated the presence of Cas9 DNA and its expression in the mesenteric lymph node and spleen obtained from the CRISPR-treated SIV-infected animals (KP43, KM77 and KV88), both exhibiting viral DNA cleavage by CRISPR, but not in the untreated control animal (KK09). In addition, we examined other lymph node tissues for Cas9 DNA expression and found distribution by DNAScope in axillary, deep cervical and colonic lymph nodes (Supplementary Fig. S11). In addition, we performed dual RNAScope for SIV and Cas9 in lymph node of KV88 and noticed a loss/lack of SIV signal in locations where the AAV9 Cas9 is located (Supplementary Fig. S12). In order to determine if AAV9/CRISPR-Cas9 was capable of viral excision in CD4+ T cells, we isolated CD4+ T cells from two SIV-infected ART-treated animals and transduced with AAV9/CRISPR-Cas9. We detected the presence of Cas9 as well as found evidence of Gag-3′LTR excision (Supplementary Fig. S13a) that was confirmed by Sanger sequencing. Dual Cas9 DNAScope followed by immunohistochemistry for CD3+ showed the presence of Cas9 DNA in T cells after in vivo administration of AAV9/CRISPR-Cas9 (Supplementary Fig. S13b).

### Editing of the SIV genome in ART-treated living animals by CRISPR

**Fig. 4:**
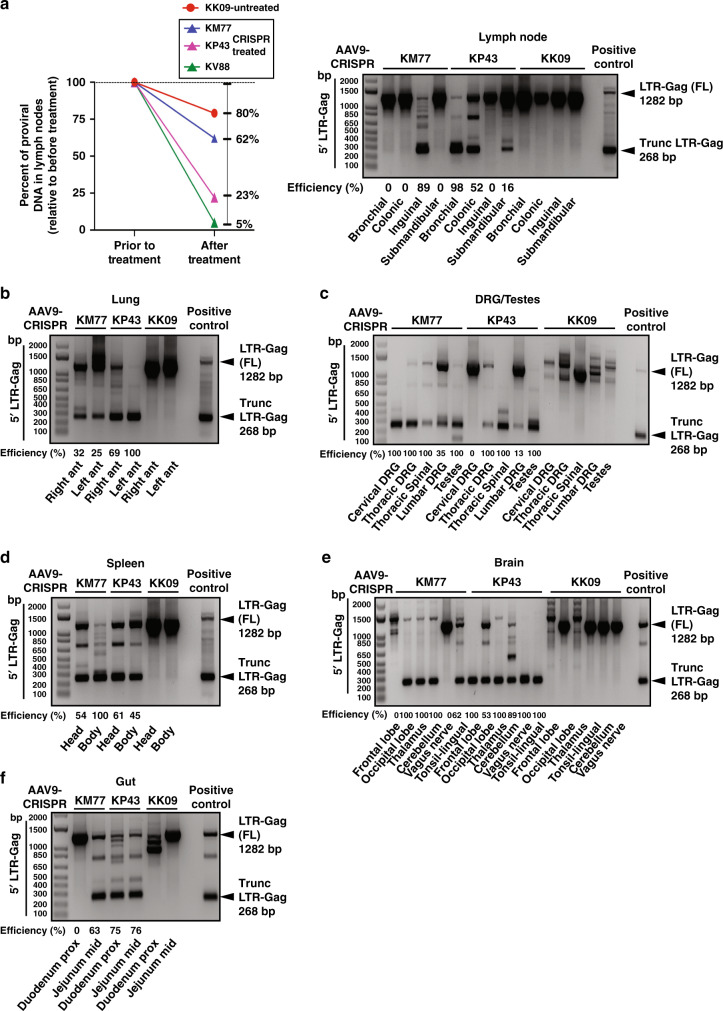
Successful SIV viral excision in spleen, lung and lymph nodes and several areas of other tissues as indicated from in vivo AAV-9-CRISPR-treated rhesus macaques. **a** ddPCR for SIV proviral DNA was performed before in vivo AAV-9-CRISPR treatment in LN biopsies and in a lymph node at necropsy. The percent decrease in SIV proviral DNA in LNs after in vivo AAV-9-CRISPR treatment was compared to SIV proviral DNA in LN biopsies prior to CRISPR. There was a greater percent decrease in KM77, KP43 and KV88 (CRISPR) vs. KK09 (control). In vivo excision was confirmed in lymph nodes of the CRISPR-treated animals by the PCR amplification and detection of the trunc 5′LTR to gag (268 bp). No excision was detected in KK09. **b**−**f** In vivo excision was confirmed in tissue (right and left lung (**b**), dorsal root ganglia (DRG), spinal cord and testes (**c**), head and body of spleen (**d**), brain and tonsil (**e**) and gut (**f**)) of the CRISPR-treated animals by the PCR amplification and detection of the trunc 5′LTR to gag (268 bp). No excision was detected in KK09. The percent excision efficiency in vivo (Efficiency (%)) shown under the PCR was calculated by quantification of the excised band (Trunc.) divided by the  sum of the  full-length band (FL) plus the excised  bands  times 100%. Source data are provided as a Source Data file.

Results from SIV ddPCR tailored for detecting SIV DNA sequence demonstrated clear decline in SIV DNA in lymph nodes (LN) after CRISPR (38−95%) compared to SIV DNA in LN biopsies performed prior to CRISPR (Fig. 4a). We also observed a mild decline (20%) in the SIV DNA of the control untreated sample that may be attributed to natural variation in viral-infected cells between LNs viral decay by ART during the course of the study. In the follow-up studies, we assessed the excision of the viral DNA by CRISPR in all collected tissues by double-nested PCR and sequencing of the amplicon(s) (Fig. 4a–f and Supplementary Figs. S6, S7). The appearance of 268 (Fig. 4a–f and Supplementary Figs. S7, S10) and 171 bp amplicons (Supplementary Figs. S6, S7, S10) suggests the removal of the viral DNA sequences corresponding to 5′ LTR to Gag gene and Gag gene to 3′ LTR, respectively, which were confirmed by DNA sequencing (Supplementary Fig. S8). Excision efficiency was determined in the 5′LTR-gag in all tissues. Of note, in some instances editing of proviral SIV DNA was associated with insertion or deletion of the unrelated DNA sequences at the breakpoints (Supplementary Fig. S9), that were most likely caused by the non-homologous end joining (NHEJ) repair pathway that is known for its infidelity in repairing damaged DNA^12,13^.

## Discussion

Soon after infection, HIV-1 genomic RNA is converted to DNA by reverse transcription, and proviral DNA is integrated into the host genome of the infected cells and serves as the template for replication of new viral genome. The full-length integrated proviral DNA may also remain in a silent but in an activatable state in some cells and tissues, most notably, memory CD4+ T cells, spleen, bone marrow, lymph node, liver, among others and serve as viral reservoirs. At this point, HIV-1 remains impervious to clearance from the host by immune cells or by current ART. As such, one can envision a scenario in which HIV-1 infection becomes reminiscent of a genetic disease whereby the reservoirs now become sanctuary sites for harboring the disease-causing gene, i.e. proviral DNA, which has been acquired by infection. Of note, once a person is diagnosed with HIV-1 infection, proviral DNA has already been positioned within the host genome and ART treatment can effectively stop the virus life cycle at multiple stages yet has no effect on the integrated proviral DNA. Thus, any interruption in ART results in rebound of viremia that most likely originates from the integrated proviral DNA in the reservoirs. As such, a permanent cure for HIV-1 disease requires, in addition to a pharmacologic approach for prevention of viral propagation and its spread, a genetic strategy to eradicate the silent proviral DNA to the extent that even in the absence of the ART no viremia is detected. One strategy to achieve this goal would be inactivation of the integrated proviral DNA and/or excision of the segments of the integrated HIV-1 DNA from the host chromosome with no collateral damage to the host genome. Earlier studies in cell models for HIV DNA latency and reactivation have demonstrated that gene editing technology involving CRISPR/Cas9 may offer the opportunity for eliminating the integrated proviral DNA^5,14–16^. These proof-of-concept observations may serve as a first step toward in vivo preclinical studies by employing a delivery system that: (i) has the capacity for carrying the CRISPR genes expressing its components that includes the Cas9 endonuclease and HIV-specific targeting guide RNAs, (gRNAs); and (ii) exhibits a broad range of functional bioavailability especially to known viral reservoirs, including T cells^17^.

Interestingly, recent studies have shown no consistent enrichment of intact or inducible proviruses in any specific T-cell subset, which reveals that a targeted approach for HIV cure based solely on T-cell memory subsets^18^ will be problematic. This finding suggests that a genetic approach that is not dependent on specific identification of the viral reservoirs is valuable. We provide evidence of SIV viral excision and bioavailability of Cas9 in multiple tissues, including those highly enriched in T cells, such as lymph nodes. In addition, we show evidence that supports the cleavage of SIV DNA in T cells and Cas9 DNA within lymphocytes.

Use of an all-in-one delivery system encompassing both the Cas9 endonuclease and the multiple gRNAs will allow the simultaneous expression of Cas9 and the gRNAs upon delivery in the target cells and facilitate the efficiency of the target DNA cleavage at multiple sites and the removal of the intervening viral DNA sequence from the host genome. It is important to note that excision of  viral DNA of either the 5′LTR to gag or the gag to 3′LTR would render the virus not capable of productive viral replication; so, the removal of one or both sequences would be effective.

Earlier studies have shown that intravenous administration of AAV9 as a viral vector for distributing CRISPR in HIV-1 transgenic mice and virally infected humanized mice resulted in the efficient and broad distribution of CRISPR in blood cells as well as several viral reservoirs including spleen and lymph node, and the removal of viral DNA from several major tissues^8,19^. In a more recent set of experiments, editing of HIV-1 proviral DNA by AAV-CRISPR in blood and several lymphoid and tissues, known as sites of latency for the integrated proviral genome in infected humanized mice, resulted in the complete clearance of replication competent virus from a total of 9 out of 23 animals in three independent studies as verified by the lack of viremia after the cessation of ART^20^.

In this proof-of-concept first-in-non-human primate study, we have provided rigorous evidence for employment of CRISPR genome editing as part of a possible HIV cure strategy, and we have demonstrated the feasibility of this treatment in vivo. We demonstrated that the i.v application of the all-in-one AAV9-CRISPR-Cas9 in monkey is well tolerated and AAV9 is broadly distributed in the cells and tissues known to be viral reservoirs, leading to cleavage of the SIV genome at the target sites within the LTR and Gag gene, and excision of the intervening sequences. This is reminiscent of our recent observations using HIV-1-infected humanized mice^20^.

AAVs are known to have multiple tropisms and delivery efficacies^21^ that lead to elimination of replication competent virus after cessation of ART^20^. As in the mouse study, here we selected AAV9 as one-time intravenous administration to facilitate expression of proteins in a broad spectrum of tissues, including CNS^22^. In this initial first-in-primate study, three animals, negative  for pre-exisiting AAV9 neutralizing antibodies, were sacrificed 3 weeks after AAV9/CRISPR-Cas9 treatment and one used as a control. We acknowledge that this study has a small cohort and that in order to do statistics we need larger numbers of animals. This is only a small-scale study but the first of its kind to show SIV viral excision in blood and tissues after a single in vivo injection of CRISPR-Cas9. Future study will examine a larger cohort and if a longer period post-infusion is necessary to develop a maximum effect. The variation in the efficiency of viral excision cannot be explained by the presence of pre-existing antibodies to AAV9. In addition, in this study, as the animals remained on ART, results from further studies will shed light on the question of whether long-term viral remission in the absence of ART is associated with elimination of virus from specific key reservoirs and whether or not if reduction in the latent cell reservoirs by CRISPR will delay or eliminate viral rebound after discontinuation of ART.

The pattern of Cas9 in lymph nodes by DNA and RNAScope is within B-cell follicles. We cannot rule out that follicular dendritic cell maybe trapping a portion of the AAV9 and forming of immune complexes. However, we have evidence to suggest that the efficiency of viral excision in lymph nodes can be as high as 100% and the change in SIV viral DNA in lymph nodes before and after CRISPR was up to 95%.

Data of Drs. Siliciano demonstrate that with ART only the half-life of viral DNA in T cells is 44 months and at that rate of decay it would take greater than 70 years for a pool of just 10^6^ cells to decay completely^23^. In our ddPCR studies in lymph nodes, we found up to a 95% (38, 77 and 95%) reduction in the intact SIV DNA in just a few short weeks, compared to 20% in the non-CRISPR-treated animal. This large percent of decrease in the SIV viral DNA cannot be due to ART alone. We agree that the sample size is small, but these data in combination with the successful viral excision shown by PCR analysis in multiple tissues and blood is evidence that CRISPR is having an impact on lower viral DNA.

In conclusion, these observations underscore the potential use of this strategy for elimination of the virus in preclinical settings and perhaps its application in the clinic based on future clinical trial outcomes in PWH. Moreover, the successful employment of gene editing methods, by simple i.v. inoculation, as tested in animal models, may offer promising steps for the development of strategies for the treatment of a broad range of human diseases caused by gene mutations including cancer simply by a one shot i.v.

## Methods

### Study approval

All experimental protocols involving the use of laboratory animals were approved by the Institutional Animal Care and Use Committees (IACUC) ensuring the ethical care and use of laboratory animals in experimental research at Tulane University. All animal studies were performed in compliance with Tulane institutional policies and NIH guidelines for laboratory animal housing and care.

### Cell culture

HEK293T cells (the American Type Culture Collection, Manassas, VA) were cultured in high glucose Dulbecco’s Modified Eagle Medium (DMEM) supplemented with 10% fetal bovine serum (FBS) and gentamicin (10 µg/ml). PBMCs were isolated from whole blood by gradient centrifugation on Ficoll-Paque for 30 min at 600 × *g* then were stimulated with PHA (5 µg/ml) for 24 h in RPMI with 10% FBS and gentamicin (10 µg/ml) supplemented with human recombinant interleukin-2 (rIL-2) at a concentration of 30 ng/ml (STEMCELL Technologies, Seattle, WA). Fresh media are exchanged every 2−3 days.

### Design of gRNAs, construction of CRISPR-Cas9 expression plasmid and the AAV9 vector

Benchling CRISPR guides designer tool (https://www.benchling.com) was used to screen SIVmac239 (GenBank: M33262.1) sequence for possible gRNA protospacer regions followed by saCas9 specific PAM: NGGRR(N). A pair of gRNAs showing the best predicted on-target (in SIVmac239 genome) and the lowest off-target (in rhesus macaque genome, NCBI assembly Mmul_8.0.1.) activities was selected: one targeting the SIVmac239 LTR promoter region and the other targeting the gag gene. Next, a pair of oligonucleotides for each target site with 5′-CACC and 3′-AAAC Bsa1 overhangs was obtained from Integrated DNA Technologies (IDT, Coralville, Iowa, Supplementary Table S1), annealed, phosphorylated and ligated into *Bsa*I digested, dephosphorylated pX601-AAV-CMV:NLS-saCas9-NLS-3xHA-bGHpA;U6:BsaI-sgRNA (a gift from Feng Zhang via Addgene) (61591; Addgene). For multiplex gRNA cloning, the U6-LTR-SIV gRNA scaffold cassette from pX601-CMV-saCas9-LTR-SIV was amplified using T795/T796 primers (Supplementary Table S1) and cloned using In-Fusion HD Cloning Kit (Clontech, Mountain View, CA) into *EcoR*I and *Kpn*I linearized pX601-CMV-saCas9-Gag-SIV plasmid resulting in pX601-CMV-saCas9-LTR/Gag-SIV AAV delivery vector. Finally, sequence verified plasmid was sent for packaging into AAV9 serotype (Vigene Biosciences Inc., Milton Park Abingdon, UK). AAV9 is chosen as the vector for CRISPR-Cas9 delivery for its robust transduction efficiencies in multiple tissues along with the central nervous system as a significant putative reservoir for SIV. The notion was to permit efficient AAV entry into all putative SIV target tissues, including the brain.

### In vitro SIVmac239 infection

HEK-293 T cells were transfected using CaPO_4_ precipitation method in the presence of chloroquine (50 µM) with 25 µg of pEcoSIVmac239-eLuc and 5 µg of pCMV-VSV-g (#8454 Addgene) /2.5 × 10^6^ cells/100 mm dish. Next day, media was replaced; and 24 and 48 h later supernatants were collected, clarified at 3000 RPM for 10 min, filtered through a 0.45 µm filter, and concentrated by ultracentrifugation for 2 h with 20% sucrose cushion. Viral pellets were resuspended in Hank’s basic salt solution (HBSS) by gentle agitation overnight and aliquoted. HEK293T cells were infected with EcoSIVmac239-eLuc/VSV-g at MOI 0.001. Forty-eight hours later cells were transduced with AAV9-SaCas9-LTR/Gag-SIV at MOI 2.5 × 10^5^ (GCs).

### Macaque screening for AAV1, AAV8 and AAV9 neutralizing antibodies

Rhesus macaques were pre-screened for AAV neutralizing antibodies before the study started. Recombinant AAV (rAAV) reporter virus was made by triple transfection of AAV9/AAV2 Rep plasmid, a helper plasmid (Cell BioLabs), and pAAV.CAG.Luciferase plasmid (Addgene #83281)^24^ at a 1:1:1 ratio in HEK293T cells using PEIpro transfection reagent (Polyplus) based on the manufacturer’s instructions. Cells were harvested after 48 h, washed twice with phosphate-buffered saline (PBS), and then treated with AAV lysis buffer (150 mM NaCl, 50 mM Tris-HCl pH 8.0, 2 mM MgCl_2_). Cells underwent three freeze-thaws in the lysis buffer. Benzonase (25 U/ml) and 0.1% Triton X-100 were added to the cell lysate which was then incubated for 1 h at 37 °C. Lysates were centrifuged at 7000 × *g* for 1 h at 10 °C and supernatant was passed through a 0.45-μm filter. AAV was purified using POROS CaptureSelect AAVX for AAV1 (or AAV8 or AAV9) columns (Thermo Fisher). Purified rAAV reporter virus was tittered on HEK293T cells for RLUs > 10,000. Serum samples from individual macaques were diluted 1:5 and then mixed 1:1 with diluted rAAV reporter virus in HEK293T expression medium in duplicate in a 96-well plate. Serum/rAAV mixes were incubated for 1 h at 37 °C. HEK293T cells were then added to the rAAV/serum mix at 3 × 10^4^ cells/well. Infection was measured after a 24-h incubation using britelite plus Reporter Gene Assay System (Perkin Elmer). Luciferase expression was quantified using a VictorNivo (Perkin Elmer). Infection was calculated by dividing the luciferase signal in samples mixed with animal serum by the luciferase signal in the absence of serum, with background signal subtracted out, and multiplied by 100. Animals were considered to be seronegative with a percent infection of >95%. All animals in this study (KT38, KR11, KP43, KK71, KM77, KK09, KV88, KP78) were AAV9-negative. KK71, KT38, KK09, KR11, KP43 were also AAV8-negative and KM77; KK71, KP43, KT38 were AAV1-negative.

### Animals used in the study and ethical statement

Animals were housed at the Tulane National Primate Research Center (TNPRC; Covington, LA). All animals used in this study were handled in strict accordance with the American Association for Accreditation of Laboratory Animal Care with the approval of the Institutional Animal Care and Use Committee of Tulane University. Eight male Chinese rhesus macaques (*Macaca mulatta*) were used in this study. In our studies, we started with eight Chinese rhesus macaque animals that were all screened for pre-existing AAV9 antibodies and were negative. All animals were inoculated intravenously with SIVmac239 (100TCID50 provided by Virus Characterization, Isolation and Production Core, Division of Microbiology, TNPRC). A daily ART regimen^25^ of Tenofovir Disoproxil Fumarate (TDF) 5.1 mg/kg, Emtricitabine (FTC) 30 mg/kg and Dolutegravir (DTG) 2.5 mg/kg was given s.q. once daily starting at 78 days post infection and continuing for the duration of the study. We performed ex vivo assessment of viral excision in PBMCs from animals to check if animals were suitable for in vivo AAV9/CRISPR-Cas9 treatment (Fig. 2b and Supplementary Fig. S1). All animals had successful excision ex vivo in blood. AAV-9 was packaged by the viral vector core at the University of North Carolina. The viral stock was 8.2 × 10^12^ genome copies (GC)/ml. We randomly assigned three (KP43, KM77 and KV88) animals to receive AAV9/CRISPR-Cas9 in vivo and one animal (KK09) that was necropsied for a nontreated control (no CRISPR). Three animals (KM77, KP43 and KV88) were given 10^13^GC/kg, i.v. (1.16 ml/kg) of AAV9 in 100 ml final volume of sterile PBS. Animals were pretreated with diphenhydramine (1 mg/kg). Infusion was delivered with a Razel listed infusion pump (824E model: A-99) at a rate of 1 ml/per min. Animals were sacrificed for tissue collection 3 weeks after AAV infusion. One animal served as the control (KK09). Animals were anesthetized with ketamine-HCL and euthanized by intravenous pentobarbital overdose. A full SIV necropsy was immediately performed and blood, CSF, snap-frozen tissues, and paraffin embedded tissues were obtained for analyses. The other four animals were used for a different study. KP78 blood was only used in Supplementary Fig. S3 for a negative control as a non-CRISPR-treated animal. This animal was not necropsied.

### Monitoring of animals on study for viral loads and clinical pathology. Histological and pathological observations

Through the entire animal study, viral load, blood chemistries, complete blood counts and coagulations panels and weight were performed to assess toxicity associated with cART and AAV/safety. Viral load was quantified in the Pathogen Detection and Quantitation Core (PDQC), Division of Microbiology, TNPRC using routine methods^26^. Blood chemistries and complete blood counts (CBCs) were performed in the clinical pathology core at TNPRC.

### Ex vivo AAV9-CRISPR-SIV treatment

A total of 10^6^ freshly isolated and PHA-stimulated PBMCs from infected animals were transduced with AAV9-SaCas9-LTR/Gag-SIV at MOI 2.5 × 10^5^ (GCs). Cells were harvested after 6 days for the analysis of CRISPR/Cas9 and gRNAs expression (RT-PCRs) and viral excision (PCR genotyping). For ex vivo AAV9/CRISPR-Cas9 treatment of CD4+ T cell (Supplementary Fig. S13), T cells were isolated from fresh blood of two SIV-infected ART-treated animals (from an ongoing study) (NHP1 and NHP2) using rhesus CD4 isolation kit (Miltenyi). After 12 days in culture, T cells were transduced with AAV9-SaCas9-LTR/Gag-SIV at MOI 2.5 × 10^5^ (GCs). Cells were harvested after 3 days and genomic DNA was extracted using the Nucleo Spin Tissue Kit (Macherey-Nagel).

### Nucleic acid extractions and standard PCR and RT-PCR assays

For viral excision testing, blood or frozen tissues sent to Temple University from the Tulane National Primate Research Center (Tulane University School of Medicine) were homogenized using BeadBlaster24 homogenizer (Benchmark, USA) using 1.5 mm zirconium beads and following settings: speed 6, time 40 s, cycle 03 and inter 30 s. T1 buffer from NucleoSpin Tissue kit (Macherey-Nagel, Duren, Germany) was used for homogenization/initial lysis followed by overnight proteinase K digestion. Extraction of genomic DNA was completed according to the protocol of the manufacturer. For standard PCRs (Supplementary Table S1), 250 ng of extracted DNA was subjected to PCR using Fail Safe PCR kit and buffer D (Epicentre, Madison, WI, USA) under the following PCR conditions: 94 °C 5 min, 25 cycles (94 °C 30 s, 55 °C 30 s, 72 °C 120 s), 72 °C 7 min using first-round primers followed by nested PCR using diluted first-round PCR reaction and subsequently followed by third-round PCR using diluted nested PCR reaction. Third-round PCR products were subjected to Sanger sequencing directly if only one amplicon population was detected by agarose gel electrophoresis. For multiple amplicons detected, in order to investigate the composition of SIV excision, each amplicon population was separated and purified from an agarose gel electrophoresis and then cloned into TA vector (Invitrogen, Carlsbad, CA, USA). Plasmid DNA containing excised SIV amplicon was purified from each bacterial colony for Sanger sequencing (Genewiz, South Plainfield, NJ, USA). For RT-PCR, TRIzol reagent (Ambion, Austin, TX, USA) was used for initial RNA extraction followed by clean-up using RNeasy kit (Qiagen, Hilden, Germany) with DNAse I digestion in the extraction column. Total 1 μg of RNA was used for M-MLV reverse transcription (Invitrogen). For gRNA expression screening specific reverse primer (pX601gRNA scaffold/R, Supplementary Table S1) was used in RT reaction followed by standard PCR using target LTR or Gag sense oligos as forward primers (Supplementary Table S1) and agarose gel electrophoresis. For checking saCas9 mRNA expression oligo-dT primer mix was used in RT and cDNA were subjected to PCR using saCas9 specific primer pairs and β-actin as a reference (Supplementary Table S1). Sanger sequencing results were analyzed using Clustal Omega (EMBL-EBI) multiple sequence alignment tool and Sequence Scanner Software 2 (Applied Biosystems).

### Excision efficiency calculations

To ensure consistent and unbiased image analysis, using Adobe Photoshop, the tiff images of agarose gels were converted to grayscale, cropped, combined side by side into a single master image and carefully aligned using DNA size markers as a reference point. Next, the gray levels were adjusted so the background became uniform across the whole master image and nonspecific, not correct size bands were removed. Finally, the intensities of remaining top and bottom bands, representing full-length and CRISPR-cleaved/end-joined truncated 5′LTR-gag amplicons, were calculated using ImageJ gel analysis tool. Briefly, profile plots were generated for each line of the agarose gels and areas of the peaks corresponding to the top and bottom bands were measured and then converted in Microsoft Excel into percent excision using formula: [bottom band peak area/top and bottom bands peak areas)] × 100%.

### ddPCR for detection of AAV vector and SIVmac239 nucleic acids

ddPCR was performed based on the water−oil emulsion droplet technology, using the ddPCR™ Supermix for Probes reagents in the QX200™ Droplet Digital™ PCR system (Bio-Rad Laboratories, Hercules, CA, USA). For quantification of AAV vector, the eluted cellular DNA was PCR amplified using Taqman set targeting the SaCas9 AAV transgene and as a reference rhesus macaque TERT gene (Supplementary Table S1). A total of 50−100 ng DNA from each tissue was used as template for ddPCR amplifications with thermal cycling conditions used 98 °C 5 min, 45 cycles (98 °C 5 min), 45 cycles (98 °C 15 s, 60 °C 30 s). For quantification of AAV vector expression 1 μl of RT-PCR reaction was used and the same SaCas9 Taqman set like for DNA analysis, as a reference 0.1 μl of reaction was used and Taqman set specific to beta-actin. Data acquisition and analysis are done using QX200 droplet reader and QuantaSoft™ software provided with the instrument.

### DNAscope and RNAscope

AAV9-delivered saCas9 DNA and RNA were visualized using DNAscope and RNAscope^26^, according to the specifications of the manufacturer (ACDBio). The probes were designed to target saCas9 DNA (Cat# 505631; ACDbio) and saCas9 RNA (Cat# 501621; ACDbio), each with 20 ZZ pairs. Slides were deparaffinized through xylene, washed in 100% ethanol, and air dried. Sections were treated with heat-induced Target Retrieval (92−100 °C) and incubated with protease (40 °C). The probe was hybridized in a humidity chamber at 40 °C for 2 h. The saCas9 DNA and RNA were detected by amplification and chromogenic development using the Alkaline Phosphatase (AP), Red Chromogen detection kit (ACDBio). Sections were counterstained with hematoxylin, dried at 60 °C, cleared with xylene, and mounted. For analysis, since the red chromogen has fluorescent emission, we used negative controls for determining the red fluorescent threshold and then applied the threshold to the slides used in the study. We then determined the area of positive staining for 30 nonoverlapping images for each slide with a Keyence BZ-X700 Microscope and generated an average positive area for the animal’s tissue section. The representative images in the figures were taken in brightfield to show localization of red staining with relevant tissue organization (follicular zones) for lymphoid tissues through the hematoxylin staining. saCas9 DNA and RNA area was quantified using Batch Fluorescent Analysis and reported as the average positive area (log_10_ transformed).

### Colocalization of saCas9 and SIV

AAV9-delivered saCas9 DNA and SIV RNA were visualized using RNAscope probes (ACDBio) and Opal 4-Color Manual IHC kit dyes (Cat# NEL810001KT; Akoya Biosciences). The probes were designed to target saCas9-O1 DNA (ACDBio) and SIVmac239 RNA (Cat# 312811; ACDBio). Tissue slides were deparaffinized through xylene for 20 min and washed in diminishing concentrations of ethanol for 10 min each (100−70%). Sections were treated with Blocking/Antibody Diluent and Antigen Retrieval 6 Buffer (AkoyaBio). AR6 was used two more times following probe amplification and opal dye steps to strip sections of ZZ probe pairs while leaving the dye unscathed. Probes were hybridized in a humidity chamber at 40 °C for 2 h. The saCas9 DNA and SIV RNA were detected by amplification using the Brown Chromogen detection kit (ACDBio) and were developed using one of two opal fluorophores: Opal 570 (Red) or Opal 520 (Green). Probes were conjugated to Opal Polymer HRP Ms+ Rb (AkoyaBio). Slides were counterstained with DAPI and mounted with Vectashield Antifade Mounting Media (Cat# H-1000-10; Vector Laboratories).

### SaCas9 and T-cell visualization

AAV9-delivered saCas9 RNA probe and CD3 T cells were visualized using an RNAscope probe (ACDBio), CD3 antibody (Cat# A0452; DAKO), and the Opal 4-color Manual IHC kit dyes (AkoyaBio). Tissue slides were deparaffinized through xylene for 20 min and washed in diminishing concentrations of ethanol for 10 min each (100−70%). Sections were treated with Blocking/Antibody Diluent and Antigen Retrieval 6 Buffer (AkoyaBio). The saCas9 probe was hybridized in a humidity chamber at 40 °C for 2 h, amplified using the Brown Chromogen detection kit (ACDBio), and dyed with Opal 570 for 10 min. Slides were treated with AR6 and stained with CD3 antibody overnight in 4 °C. The primary antibody was conjugated to Opal Polymer HRP Ms+ Rb (AkoyaBio), dyed with Opal 520 for 10 min, then treated with AR6. Sections were counterstained with DAPI and mounted with Vectashield Antifade Mounting Media (Vector Laboratories).

### SIV proviral DNA

Two Taqman primer/probe sets were designed: one specific to packaging signal (nucleotides 1237-1256, FAM-probe, Supplementary Table S1) and other to RRE region (nucleotides 8443−8462, HEX-probe, Supplementary Table S1). Additionally, an unlabeled allelic discrimination probe containing two GG → AG mutations was designed for RRE sequence to account for common APOBEC3G induced hypermutations in this region. APOBEG3G is a host cell defense enzyme, which edits retroviral reverse transcripts causing damage to open reading frames of the virus and degradation of viral DNA^4^. RRE carries two adjacent TGGG consensus binding sites for APOBEC3G. Five hundred nanograms of genomic DNA extracted from the lymph nodes by ddPCR using same conditions as in ddPCR section above. FAM only positive droplets represent 3′-deleted and/or hypermutated, HEX only represent 5′-deleted and double FAM + HEX positive represent intact proviral SIVmac239 genomes. Negative droplets represent lack of viral sequences or completely (3′ and 5′) deleted viruses.

### Off-target assessment

Potential off-target sites were analyzed using bioinformatic tools and the top five off-target sites for LTR and gag gRNAs were analyzed. For each off-target site, primers (Supplementary Table S1) were designed to amplify the chromosome region surrounding the off-target site. Fifty nanograms of genomic DNA isolated from ex vivo PBMCs from three different rhesus macaque animals, KK09, KM77 and KP43, untransduced and transduced with AAV9_SaCas9_LTR_gag, was subjected to PCR using Terra™ PCR Direct Polymerase kit from Takara. Two steps PCR was carried out as follow: initial denaturation at 98 °C for 2 min, then 40 cycles of denaturation at 98 °C for 10 s and extensions at 68 °C for 30 s. Amplified products were resolved on a 1% DNA-agarose gel, bands of interested were gel-purified using QIAquick Gel Extraction Kit from Qiagen and analyzed by sequencing at Genewiz. Comparison between gDNA from untransduced vs. transduced PBMCs was analyzed for excluding mutations in off-target sites. (Supplementary Table S2).

### Reproducibility

All PCR analyses were conducted in duplicate by two different operators and all PCR bands shown in the figures were sequenced and the full sequences are provided in the data source file. Representative images were repeated independently with similar results and quantitated as shown in bar graphs.

### Reporting summary

Further information on research design is available in the Nature Research Reporting Summary linked to this article.

## Supplementary information

Supplementary Information

Reporting Summary

## Data Availability

The data that support the findings of this study are available from the corresponding authors upon reasonable request. Source data are provided with this paper.

## Source data

Source Data
