# Supplementary material for: CRISPR based editing of SIV proviral DNA in ART treated non-human primates
Source: Nat Commun. 2020 Nov 27;11:6065. doi: 10.1038/s41467-020-19821-7 (PMC7695718; doi:10.1038/s41467-020-19821-7)
Supplement: Supplementary file 1 — Supplementary Information [file 41467_2020_19821_MOESM1_ESM.pdf]

October 14, 2020

CRISPR based editing of SIV proviral DNA in ART treated non-human primates

SUPPLEMENTARY MATERIAL

Pietro Mancuso[1], Chen Chen[1], Rafal Kaminski[1], Jennifer Gordon[1], Shuren Liao[1], Jake A. Robinson[1], Mandy D. Smith[1], Hong Liu[1], Ilker K. Sariyer[1], Rahsan Sariyer[1], Tiffany A. Peterson[2], Martina Donadoni[1], Jaclyn B. Williams[2], Summer Siddiqui[2], Bruce A. Bunnell[2,3,4,5], Binhua Ling[2,6,7*], Andrew G. MacLean[2,3,5*], Tricia H. Burdo[1*], and Kamel Khalili[1*]

1. Department of Neuroscience, Center for Neurovirology, Lewis Katz School of Medicine at Temple University, 3500 N. Broad Street, 7th Floor, Philadelphia, PA 19140

2. Division of Comparative Pathology, Tulane National Primate Research Center, Covington, LA 70433

3. Tulane Brain Institute, Tulane University, New Orleans, LA 70118

4. Department of Pharmacology, Tulane University School of Medicine, New Orleans, LA 70112

5. Department of Microbiology, Immunology and Genetics, University of North Texas Health Science Center, Fort Worth, TX, 76107

6. Department of Microbiology & Immunology, Tulane University School of Medicine, New Orleans, LA 70112

7. Texas Biomedical Research Institute, San Antonio, TX 78227

*Corresponding authors
Kamel Khalili, phone: 215-707-4500, Email: kamel.khalili@temple.edu
Tricia H. Burdo, phone: 215-707-1618, Email: tricia.burdo@temple.edu
Andrew G. MacLean, phone: 985-871-6489, Email: amaclean@tulane.edu
Binhua Ling, phone: 210-258-9186, Email: bling@txbiomed.org

Supplementary Material

Supplementary Tables and Figures

Supplementary Table 1. PCR primers and probes

PCR	Primer name	Coordinates	Sequence (5'→3')
**1. Standard PCRs**			
5'LTR-gag	1st round LTR F	338-357 *	GGCAGGATTACACCTCAGGA
	1st round gag R	1927-1946 *	TCTGCAGCCTCCTCGTTTAT
	2nd/3rd round 5'LTR F	377-396 *	AGACATTTGGCTGGCTATGG
	2nd round gag R	1836-1855 *	GGGTGCAACCTTCTGACAGT
	3rd round gag R	1632-1658 *	GTTTCTGTTGTTCCTGTTTCCACCACT
Gag-3'LTR	1st round gag F	1369-1388 *	CTACGACCCAACGGAAAGAA
	1st round 3'LTR R	10508-10527 *	ATTTTCCTGCTTCGGTTTCC
	2nd/3rd round gag F	1505-1524 *	CATTAGTGCCAACAGGCTCA
	2nd round 3'LTR R	10290-10309 *	CACCCAGGCTCTACCTGCTA
	3rd round 3'LTR R	10099-10122 *	AGCGAGTTTCCTTCTTGTCAGCCA
**2. Taqman ddPCRs/RT-ddPCRs**			
AAV vector SaCas9 transgene	SaCas9 F	1799-1818 **	CGACATCAAGGACATTACCG
	SaCas9 R	1873-1882 **	GCTGCTCTGGTAGATGGTCA
	SaCas9 probe FAM	1839-1858 **	AACGCCGAGCTGCTGGATCA
Rhesus macaque DNA reference	RmTERT F	Ch6 1158407-1158427 ***	GAGCTGAGATTGTGCCCTTG
	RmTERT R	Ch6 1158579-1158598 ***	CCATTTGCTGTCCTCTGCTC
	RmTERT probe HEX	Ch6 1158531-1158552 ***	CCAGCACAGATCCTGGTCCCGT
Rhesus macaque RNA reference	Rm b-actin F	Ch3 39509713-39509733 ***	GCTCTCTTCCAACCTTCCTTC
	Rm b-actin R	Ch3 39509891-39509912 ***	CGTACAGGTCTTTACGGATGTC
	Rm b-actin probe HEX	Ch3 39509837-39509860 ***	AGTTTCGTGGATGCCACAGGACTC
SIVmac239 proviral DNA	Ψ F	1162-1181 *	ACGACGGAGTGCTCCTATAA
	Ψ R	1299-1318 *	TCACGCCCATCTCCCACTCT
	Ψ probe FAM	1237-1256 *	TTGTGTTGCACTTACCTGCA
	RRE F	8406-8425 *	CTACTGGTGGCACCTCAAGA
	RRE R	8498-8515 *	AGCGGTCAGCGTCAACGA
	RRE probe HEX	8443-8462 *	TGTGCTAGGGTTCTTGGGTT
	RRE/hypermutation probe	8443-8462 *	TGTGCTAAGGTTCTTAGGTT
**3. Standard RT-PCRs**			
SaCas9	SaCas9 F	1272-1294 **	CTGGAACGGCTGAAGAAAGACGG
	SaCas9 R	1794-1818 **	CGGTAATGTCCTTGATGTCGTGGTA
gRNAs	gRNA LTR F	541-563 *<br>10002-10023 *	GAGGCATATGTTAGATACCCAG
	gRNA Gag F	1556-1576 *	GCGTCATCTGGTGCATTCACG
	pX601gRNAscaffold R	4675-4694/5048-5067 **	CGCCAACAAGTTGACGAGAT
Rhesus macaque reference	b- actin F	Ch3 39508761-39508781 ***	CTACAATGAGCTGCGTGTGGC
	b-actin R	Ch3 39509455-39509475 ***	CAGGTCCAGACGCAGGATGGC
**4. Ex vivo OFF target analysis**			
	LTR/OFF1 F	Ch1 165929357-165929376***	GCCAACCTAGGCAGTTCAGA
	LTR/OFF1 R	Ch1 165929655-165929674***	CAAGTCATGCAGTGGCAAGT
	LTR/OFF2 F	Ch7 165078305-165078324***	CCTCCTTGTGCTCTCAGGTC

	LTR/OFF2 R	Ch7 165078980-165079000***	CAACAGTTGGCATTTCCATTT
	LTR/OFF2/KP43 F	Ch3 165078836-165078855***	GAGGGGACATGGACCCTAAT
	LTR/OFF2/KP43 R	Ch3 165079125-165079144***	CAAACGCATGATCAAGGATG
	LTR/OFF3 F	Ch3 134437624-134437643***	ACCTGGTGGTTTACCTGGTG
	LTR/OFF3 R	Ch3 134437966-134437985***	ACACATTCTGCCAAACGACA
Gag OFF targets	Gag/OFF1 F	Ch15 92495549-92495567***	GCAGTGTCGTGCTCACTCAT
	Gag/OFF1 R	Ch15 92495980-92495999***	CATGAAAGACGTTGGTGGTG
	Gag/OFF2 F	Ch9 60802578-60802597***	GTAGGCCTGTGTGGTCTCGT
	Gag/OFF2 R	Ch9 60803010-60803029***	GGGGACCATAAGACCCTCAT
	Gag/OFF3 F	Ch9 127982400-127982419***	GTCCACATCCCTCTCCTGAA
	Gag/OFF3 R	Ch9 127982853-127982872***	AGCAGCTGTAAGGCGTGAAT
**5. Cloning gRNAs protospacers into pX601 AAV vector**			
Cloning single gRNAs into pX601 vector	LTR gRNA top	N/A - cloning primers	CACCGAGGCATATGTTAGATACCCAG
	LTR gRNA bottom	N/A - cloning primers	AAACCTGGGTATCTAACATATGCCTC
	Gag gRNA top	N/A - cloning primers	CACCGCGTCATCTGGTGCATTCACG
	Gag gRNA bottom	N/A - cloning primers	AAACCGTGAATGCACCAGATGACGC
Multiplexing gRNA cassettes	InFusion/T795/F	N/A - cloning primers	ATTACGCTTAAGAATTCCTAGAGC
	InFusion/T796/R	N/A - cloning primers	*GGAAATAGGCCCT*CAGACTAGGGGTTCCTGCGGCCGCAAA

* Simian (macaque) immunodeficiency virus, isolate 239, GenBank: M33262.1
** pX601 (backbone: Addgene #61591)
*** Rhesus macaque reference genome: NCBI *Macaca mulatta* Mmul_8.0.1

Supplementary Table 2. Potential off target sites in the rhesus macaque genome for SIVmac239 target LTR and Gag.

Table 2A. sivLTR B ((+)AGGCATATGTTAGATACCCAG)

Sequence	PAM	Score	Chromosome	Strand	Position	Mismatches	On-target	Verified KM77	Verified KK09	Verified KP43
ATGCATATTTTAGATCCCCAG	TTGAA	1	chr9:-36728214	-1	36728214	3	FALSE	*	*	*
CAGAATATTGTAGATACCCAG	AGGAA	0.9	chr1:-169663551	-1	169663551	5	FALSE	*	*	*
TAATATATGTTAGATACCCAT	TTGAA	0.9	chr7:+166106392	1	166106392	5	FALSE	*	*	*
AGAAATATGTGTGATACCCAG	GTGAA	0.8	chr3:-146443697	-1	146443697	4	FALSE	*	*	*
CACCATATGTAAGATACACAG	GAGAG	0.7	chr2:-57321646	-1	57321646	5	FALSE	*	*	*

Table 2B. sivGag ((+)GCGTCATCTGGTGCATTCACG)

Sequence	PAM	Score	Chromosome	Strand	Position	Mismatches	On-target	Verified KM77	Verified KK09	Verified KP43
ACAGCATCTGATGCATTCACA	GGGAG	0.8	chr15:-84990236	-1	84990236	5	FALSE	*	*	*
TCGGGATCTGTGGCATTCACG	ATGAA	0.8	chr9:+65434122	1	65434122	5	FALSE	*	*	*
TGGTCATCTGGTGCATTGACC	TAGAG	0.8	chr19:-10211821	-1	10211821	4	FALSE	*	*	*
ACCTCTTCTGATTCATTCACG	TCGAA	0.8	chr9:-131399501	-1	131399501	5	FALSE	*	*	*
AGGACATCTGGGGCATTCACA	GGGAG	0.5	chr9:-29813659	-1	29813659	5	FALSE	*	*	*

Potential off target sites for SIVmac239 LTR and Gag in rhesus macaque genome were analyzed using CRISPR design tool (Benchling.com) bioinformatic tool. The five top off target sites were chosen for each gRNA and analyzed using gDNA extracted from ex vivo PMBCs of three different animals and untransduced or transduced with AAV6-LTR-Gag. Analysis on sequencing verified the absence of mutations in transduced group.

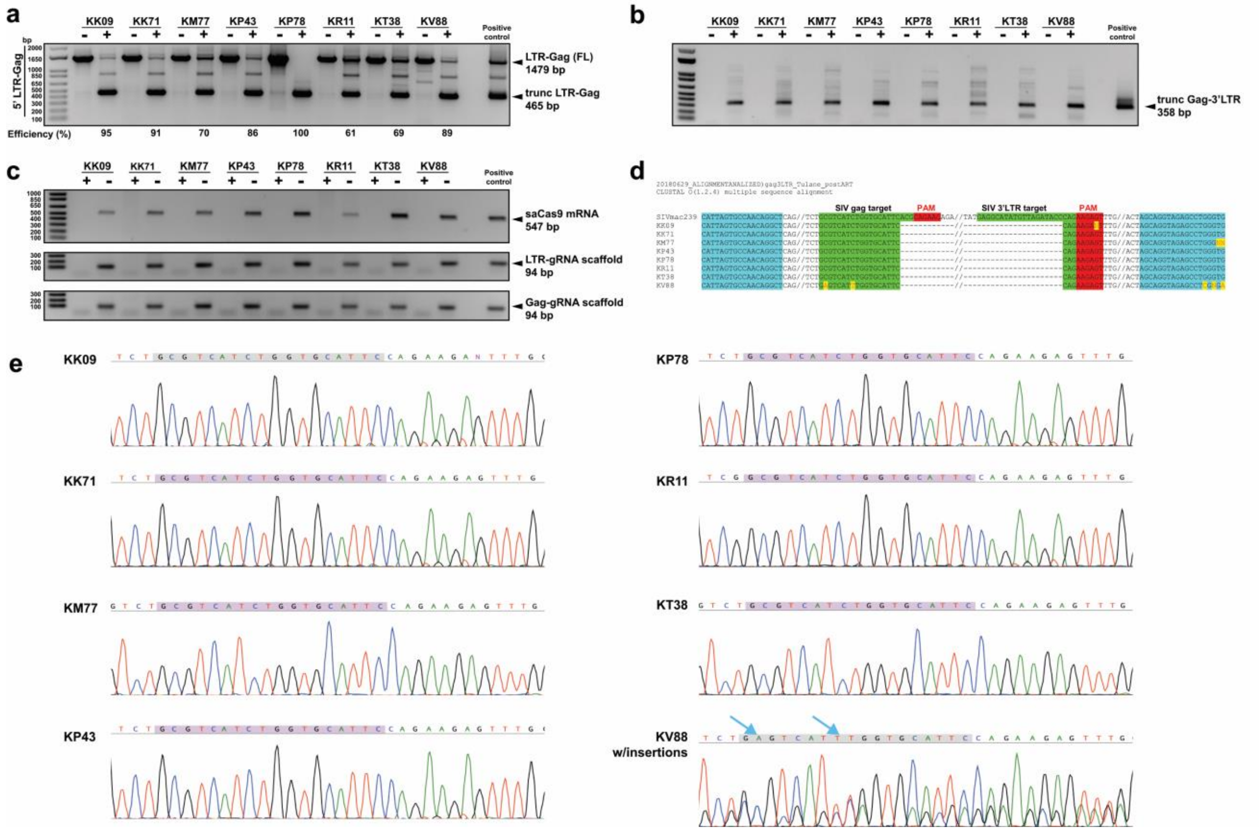

Supplementary Figure 1. Successful cleavage of SIV genome and verification of Cas9 mRNA and gRNA expression in *ex vivo* AAV-9-CRISPR treated rhesus PBMCs. AAV9-mediated delivery of CRISPR/Cas9 to PBMCs from SIVmac239-infected animals was able to excise SIV proviral DNA *ex vivo*. **a.** Truncated 5'LTR-gag (465 bp) amplicons were detected in lanes with AAV-9 CRISPR/Cas9 (+) but not in the untransformed (-). The percent excision efficiency ex vivo (Efficiency (%)) shown under the PCR was calculated by quantificationof the excised band (Trunc.) divided by the full-length band (FL) times 100 percent. **b.** Truncated gag-3'LTR (358 bp) amplicons were detected in lanes with AAV-9 CRISPR/Cas9 (+) but not in the untransformed (-). **c.** Expression was verified by the presence of SaCas9 mRNA (547 bp), LTR and Gag gRNA scaffolds (94 bp). **d-e.** Sanger sequencing was used to verify excision. The breakpoint of the viral DNA, where the truncated Gag is joined to the residual of the 3'LTR after the removal of the 8803bp DNA is shown as a dotted line. Full sequencing data are available in the source file data provided with this paper. Source data are provided as a Source Data file.

a

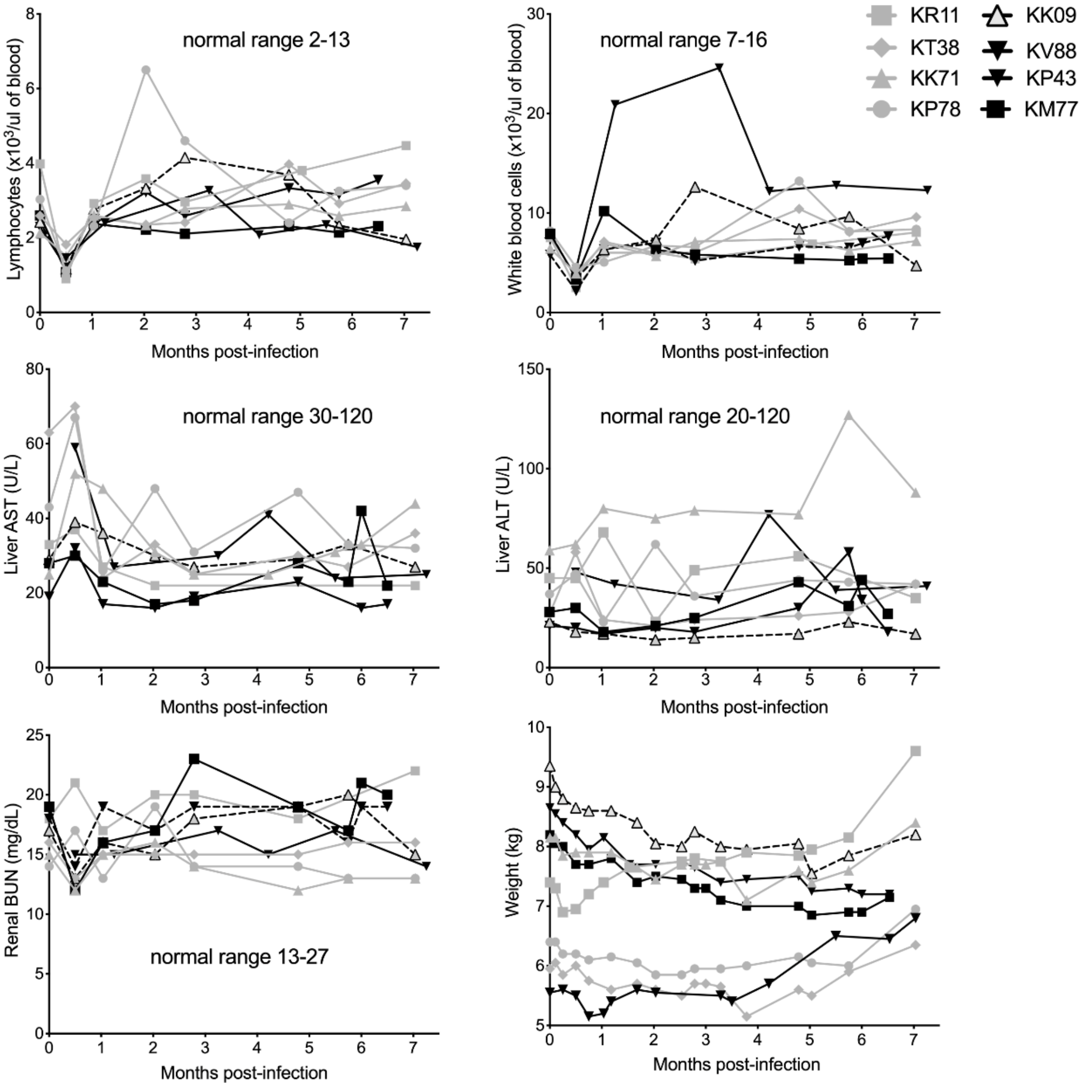
normal range 2-13
Lymphocytes (x10^3/ul of blood)
Months post-infection
normal range 7-16
White blood cells (x10^3/ul of blood)
KR11
KT38
KK71
KP78
KK09
KV88
KP43
KM77
normal range 30-120
Liver AST (U/L)
normal range 20-120
Liver ALT (U/L)
Renal BUN (mg/dL)
normal range 13-27
Weight (kg)

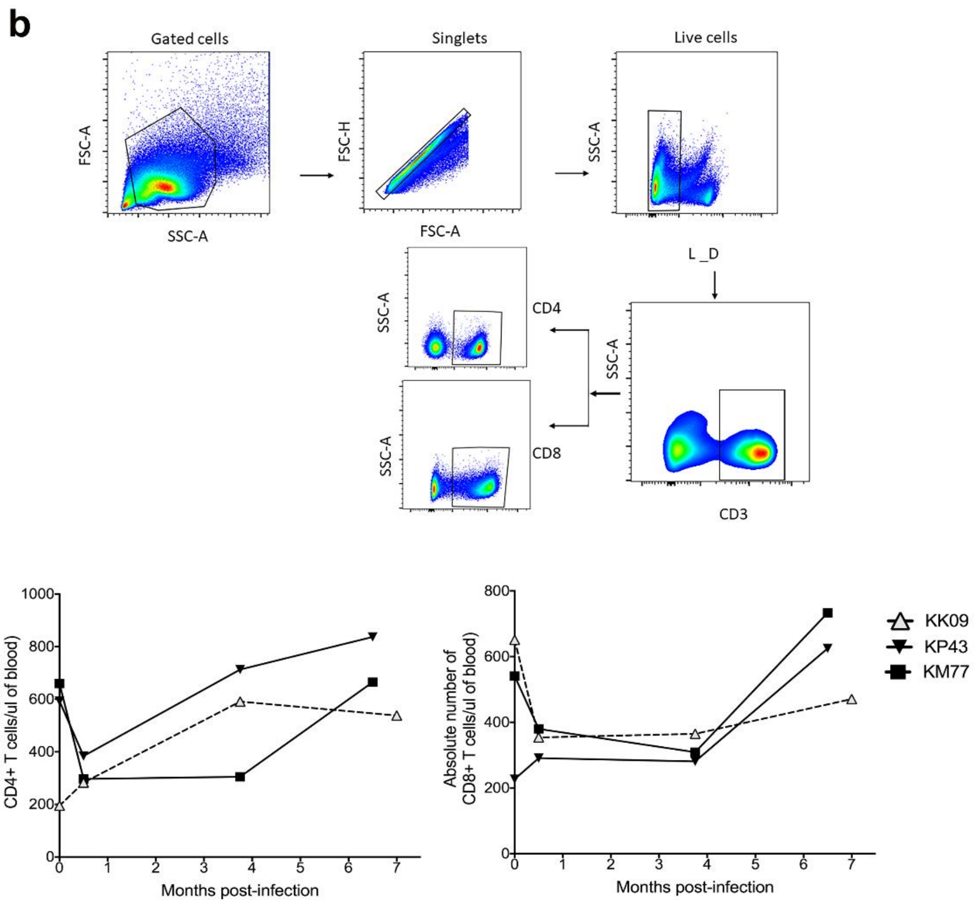

Supplementary Figure 2: Blood chemistries, complete blood counts, weights and CD4 and CD8 T cell counts by flow cytometry in the monkeys. a. Through the entire animal study, blood chemistries, complete blood counts and coagulations panels and weight were performed to assess toxicity associated with AAV/safety. There were no adverse effects from the ART or from the AAV9 inoculations. At necropsy, animals were saline perfused and a full tissue harvest was performed. No significant findings were noted on gross examination of KP43, KV88 or KK09. KM77 had enlarged mesenteric (~4x normal size) and axillary (~2x normal size) lymph nodes. Routine histologic evaluation of the spleen, lymph nodes (deep cervical, axillary, and mesenteric), liver, small intestine (jejunum and ileum), and brain (frontal lobe, basal ganglia, and hippocampus) was performed on all four monkeys. The following histological changes were noted in all three monkeys unless otherwise specified. Splenic white pulp ranged from mildly to markedly

hyperplastic consisting of expansion of the periarteriolar lymphoid sheaths (PALS) and marginal zones with occasional merging of PALS. Lymph nodes had mild to marked follicular and paracortical hyperplasia with marked sinus histiocytosis. Histiocytes within the medullary sinuses exhibited occasional erythrophagocytosis or contained intracytoplasmic brown, granular pigment (suspected hemosiderin). KK09, KP43 and KK71 had mild to moderate infiltration of eosinophils within the medullary sinuses of the mesenteric lymph node. Scattered throughout the hepatic parenchyma were clusters of immature erythroid and myeloid precursor cells (extramedullary hematopoiesis; EMH). The lamina propria of the jejunum and ileum had mild infiltration of eosinophils. In addition to eosinophilic infiltration, within jejunal crypts, KK71 had rare, adult nematodes. Randomly scattered throughout the jejunal lamina propria were rare, small to medium lymphoid nodules. Peyer's patches were noted throughout sections of ileum. No evidence of AAV-CRISPR/Cas9-related drug toxicity was noted in the tissues examined. Lymphoid hyperplasia was seen in KK09, which is a common finding in SIV-infected NHPs. **b.** Cells were gated by forward (FSC) vs side scatter (SSC), then as singlets, live cells (L_D, Live_Dead), then CD3+ T cells and then by CD4+ or CD8+T cells. Absolute number of CD4+ and CD8+ T cells over the course of infection for KK09, KP43 and KM77. Source data are provided as a Source Data file.

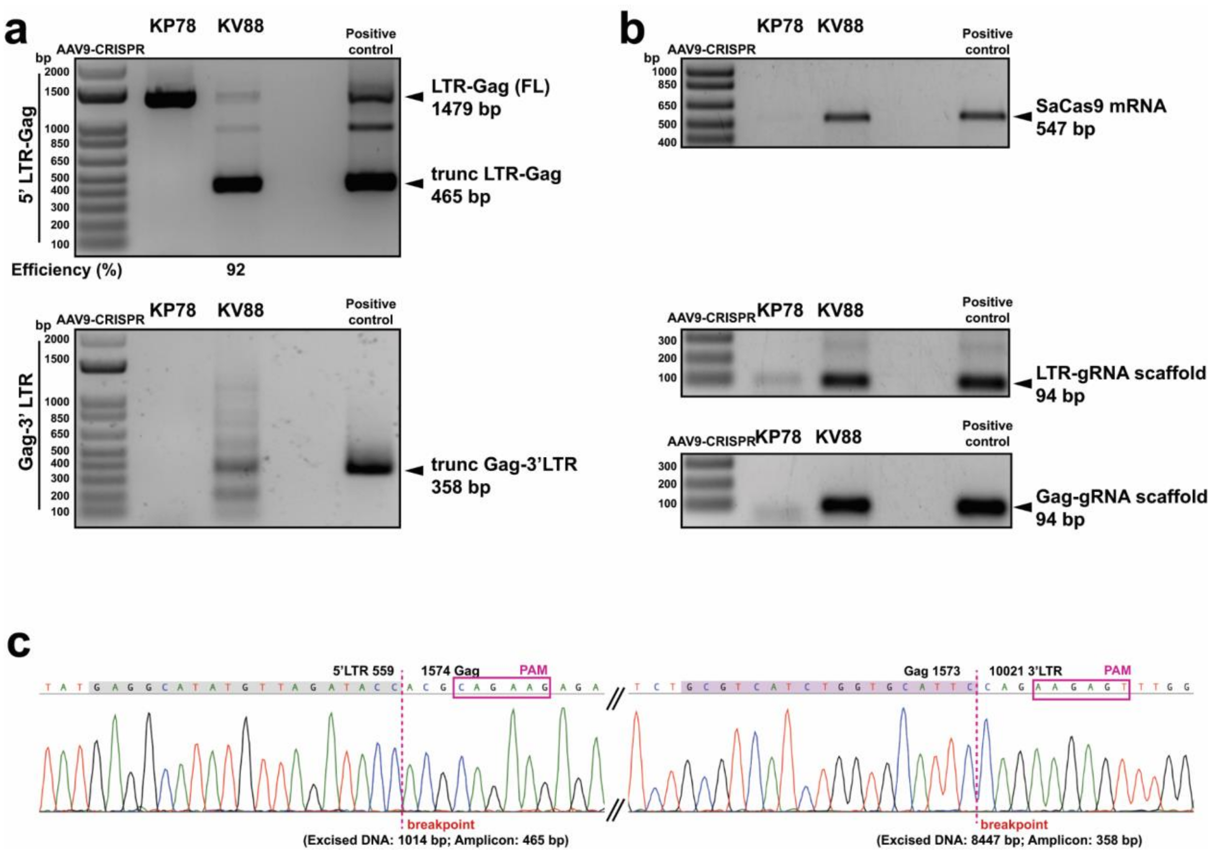

Supplementary Figure 3. In vivo editing of viral DNA by CRISPR in blood harvested from SIV-infected macaque KV88. a. *In vivo* excision of SIV DNA was confirmed in the blood of KV88 by the single-nested PCR amplification and detection of the trunc 5'LTR to gag (465 bp) and the gag-3'LTR (358 bp). Blood from SIV-infected ART treated animal, KP78, was used a no CRISPR control. The percent excision efficiency in vivo (Efficiency (%)) shown under the PCR was calculated by quantification of the excised band (Trunc.) divided by the full-length band (FL) time 100 percent. **c.** Expression was verified by the presence of SaCas9 mRNA (547 bp), LTR and Gag gRNA scaffolds (94 bp). **c.** Representative Sanger sequence tracings of 5'LTR-Gag (left) and Gag-3'LTR (right) CRISPR-Cas9 induced truncated SIV specific amplicons. Target sites are highlighted in green, PAMs motifs in red, the double cleaved/end-joined site is shown as a breaking point in red. Full sequencing data are available in the source file data provided with this paper. Source data are provided as a Source Data file.

DNA

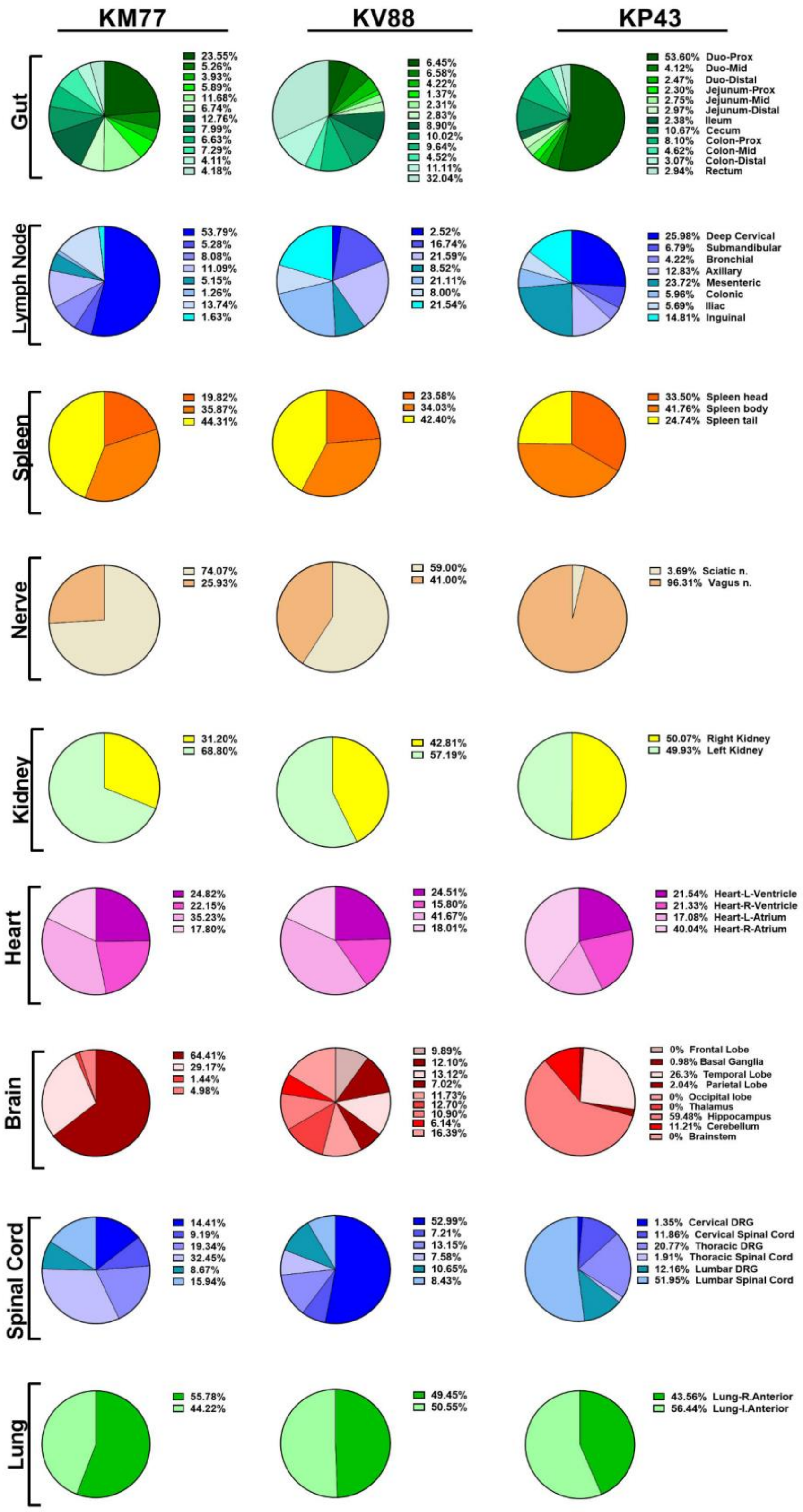

KM77
KV88
KP43
Gut
23.55% 5.26% 3.93% 5.89% 11.68% 6.74% 12.76% 7.99% 6.63% 7.29% 4.11% 4.18%
6.45% 6.58% 4.22% 1.37% 2.31% 2.83% 8.90% 10.02% 9.64% 4.52% 11.11% 32.04%
53.60% Duo-Prox
4.12% Duo-Mid
2.47% Duo-Distal
2.30% Jejunum-Prox
2.75% Jejunum-Mid
2.97% Jejunum-Distal
2.38% Ileum
10.67% Cecum
8.10% Colon-Prox
4.62% Colon-Mid
3.07% Colon-Distal
2.94% Rectum
Lymph Node
53.79% 5.28% 8.08% 11.09% 5.15% 1.26% 13.74% 1.63%
2.52% 16.74% 21.59% 8.52% 21.11% 8.00% 21.54%
25.98% Deep Cervical
6.79% Submandibular
4.22% Bronchial
12.83% Axillary
23.72% Mesenteric
5.96% Colonic
5.69% Iliac
14.81% Inguinal
Spleen
19.82% 35.87% 44.31%
23.58% 34.03% 42.40%
33.50% Spleen head
41.76% Spleen body
24.74% Spleen tail
Nerve
74.07% 25.93%
59.00% 41.00%
3.69% Sciatic n.
96.31% Vagus n.
Kidney
31.20% 68.80%
42.81% 57.19%
50.07% Right Kidney
49.93% Left Kidney
Heart
24.82% 22.15% 35.23% 17.80%
24.51% 15.80% 41.67% 18.01%
21.54% Heart-L-Ventricle
21.33% Heart-R-Ventricle
17.08% Heart-L-Atrium
40.04% Heart-R-Atrium
Brain
64.41% 29.17% 1.44% 4.98%
9.89% 12.10% 13.12% 7.02% 11.73% 12.70% 10.90% 6.14% 16.39%
0% Frontal Lobe
0.98% Basal Ganglia
26.3% Temporal Lobe
2.04% Parietal Lobe
0% Occipital lobe
0% Thalamus
59.48% Hippocampus
11.21% Cerebellum
0% Brainstem
Spinal Cord
14.41% 9.19% 19.34% 32.45% 8.67% 15.94%
52.99% 7.21% 13.15% 7.58% 10.65% 8.43%
1.35% Cervical DRG
11.86% Cervical Spinal Cord
20.77% Thoracic DRG
1.91% Thoracic Spinal Cord
12.16% Lumbar DRG
51.95% Lumbar Spinal Cord
Lung
55.78% 44.22%
49.45% 50.55%
43.56% Lung-R.Anterior
56.44% Lung-l.Anterior

Supplemental Figure 4. Biodistribution of the CRISPR-Cas9 vector (DNA) in tissues. ddPCR analysis of Cas9 transgene DNA levels in genomic DNA extracted from various tissues of SIV-infected AAV9-CRISPR-treated animals (KM77, KV88 and KP43). The percentages were calculated as the amount of Cas9 DNA in a specific tissue compartment divided by the total Cas9 DNA in that entire tissue type examined multiplied by 100. Source data are provided as a Source Data file.

RNA

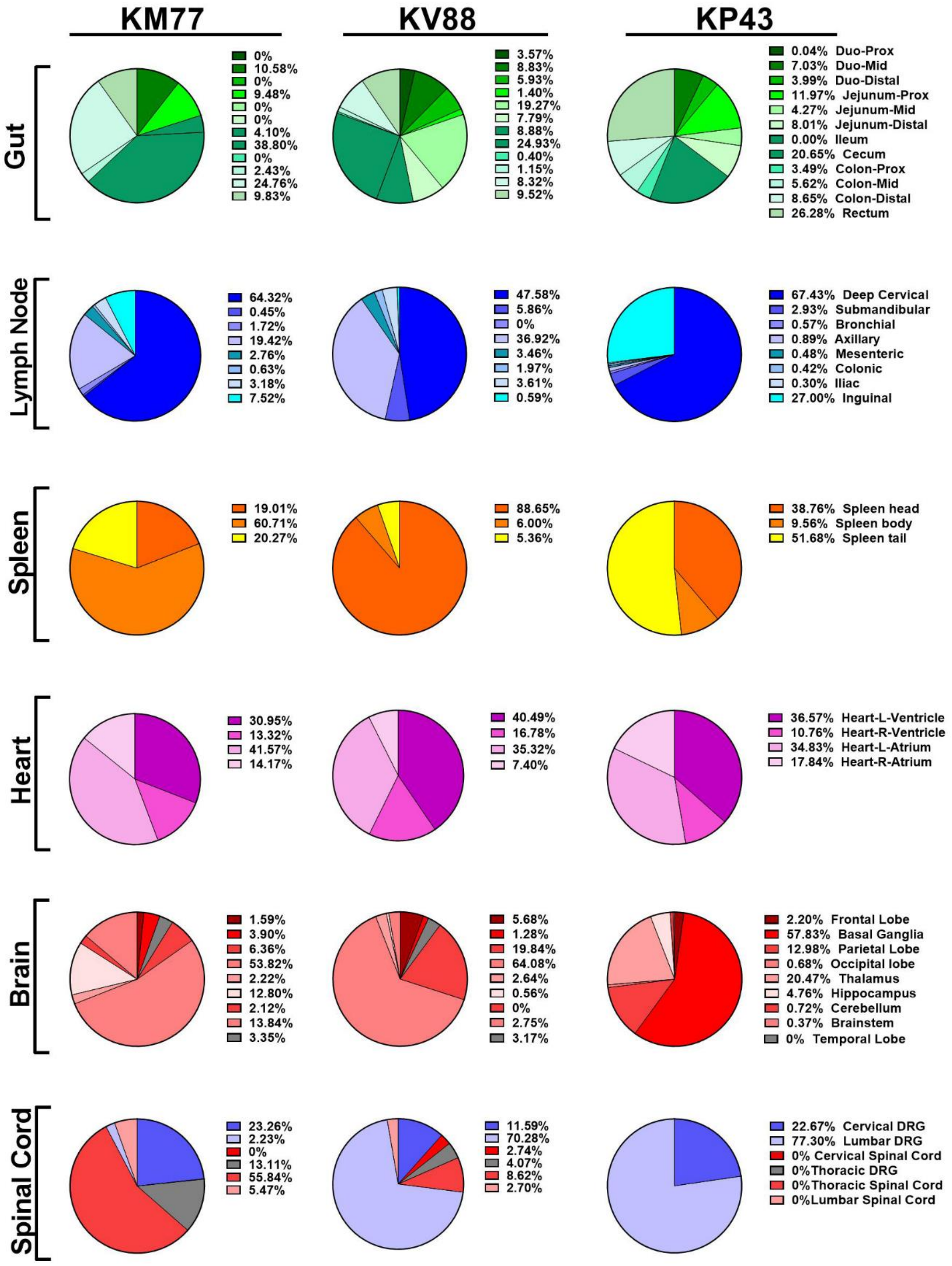

Supplemental Figure 5. Biodistribution of the Cas9 RNA in tissues. Cas9 RNA levels from various tissues of SIV-infected AAV9-CRISPR-treated animals (KM77, KV88 and KP43). The percentages were calculated as the amount of Cas9 RNA in a specific tissue compartment divided

by the total Cas9 RNA in that entire tissue type examined multiplied by 100. Source data are provided as a Source Data file.

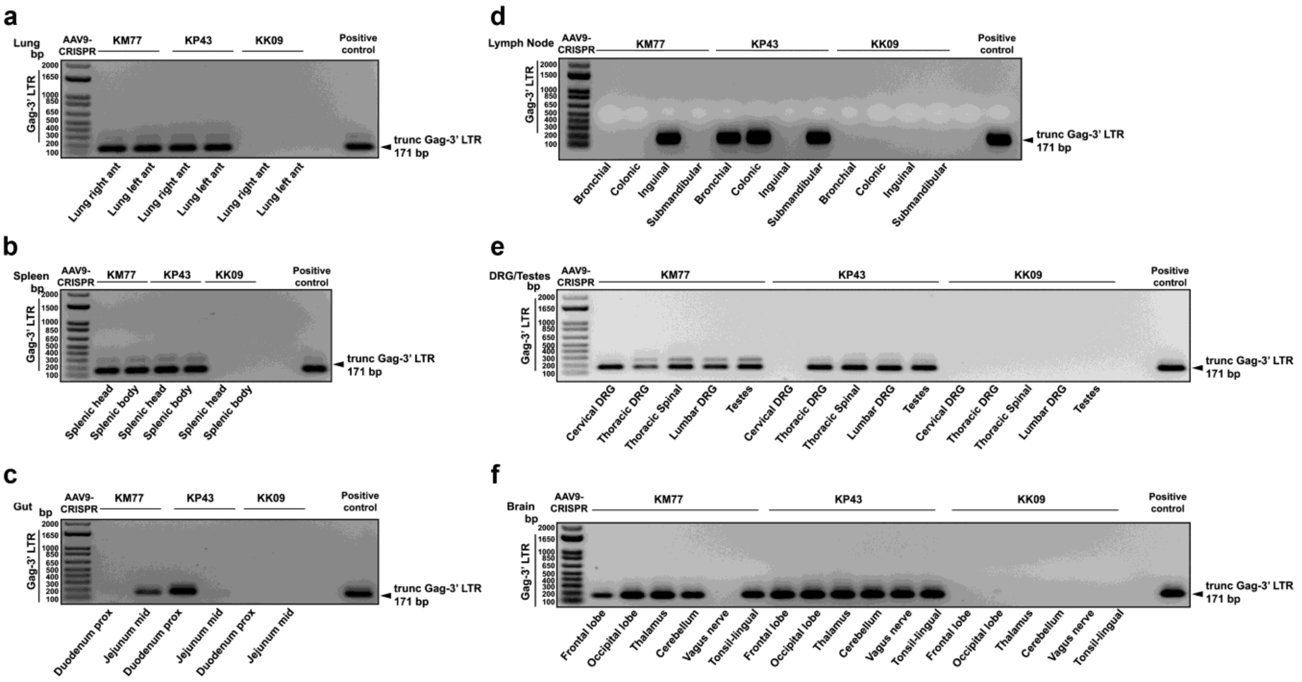

Supplementary Figure 6. ***In vivo*** **excision in tissues from SIV-infected, ART-treated rhesus macaques 3 weeks after single i.v. administration of AAV9/CRISPR-Cas9.** Rhesus macaques were infected with SIVmac239 and treated with daily ART. In the initial attempts, KM77 and KP43 animals were selected for in vivo treatment with a single i.v. infusion of AAV9/CRISPR-Cas9. *In vivo* excision of proviral DNA (Gag-3'LTR) was confirmed in the lung (a), spleen (b), gut (c) lymph nodes (d), DRG and testes (e) and brain and tonsil (f) of the animals by the double-nested PCR amplification and detection of the gag-3'LTR (171bp). No excision was detected in any tissue for the control animal KK09. Source data are provided as a Source Data file.

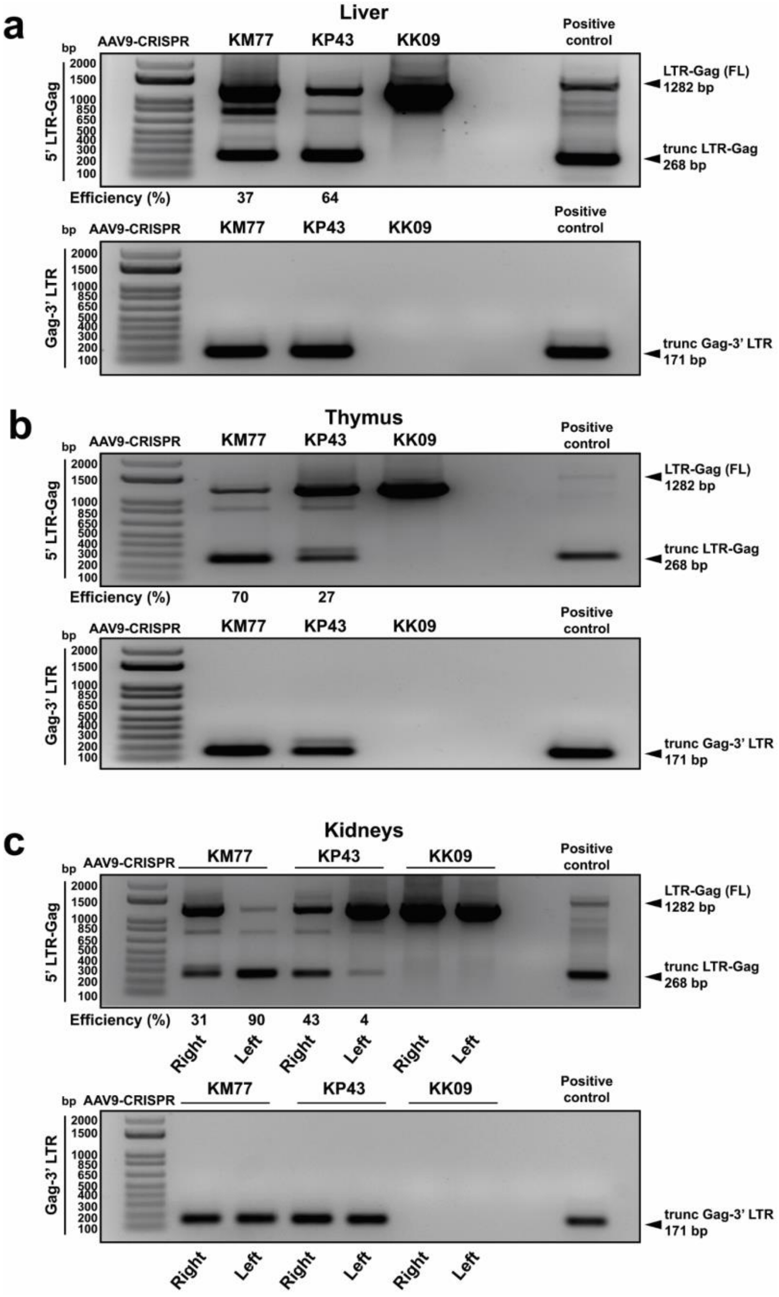

Supplementary Figure 7. CRISPR-Cas9 excision of SIV in liver, thymus and kidney. Gel electrophoresis analysis of PCR products of liver **(a)**, thymus **(b)**and kidneys **(c)** DNA of 268 bp DNA amplification after excision of the DNA fragments between 5'LTR-Gag and 171 bp DNA amplification after excision of the DNA fragments between Gag-3' LTR. The percent excision efficiency in vivo (Efficiency (%)) shown under the PCR was calculated in the 5' LTR-Gag PCR

by quantification of the excised band (Trunc.) divided by the full-length band (FL) times 100 percent. Source data are provided as a Source Data file.

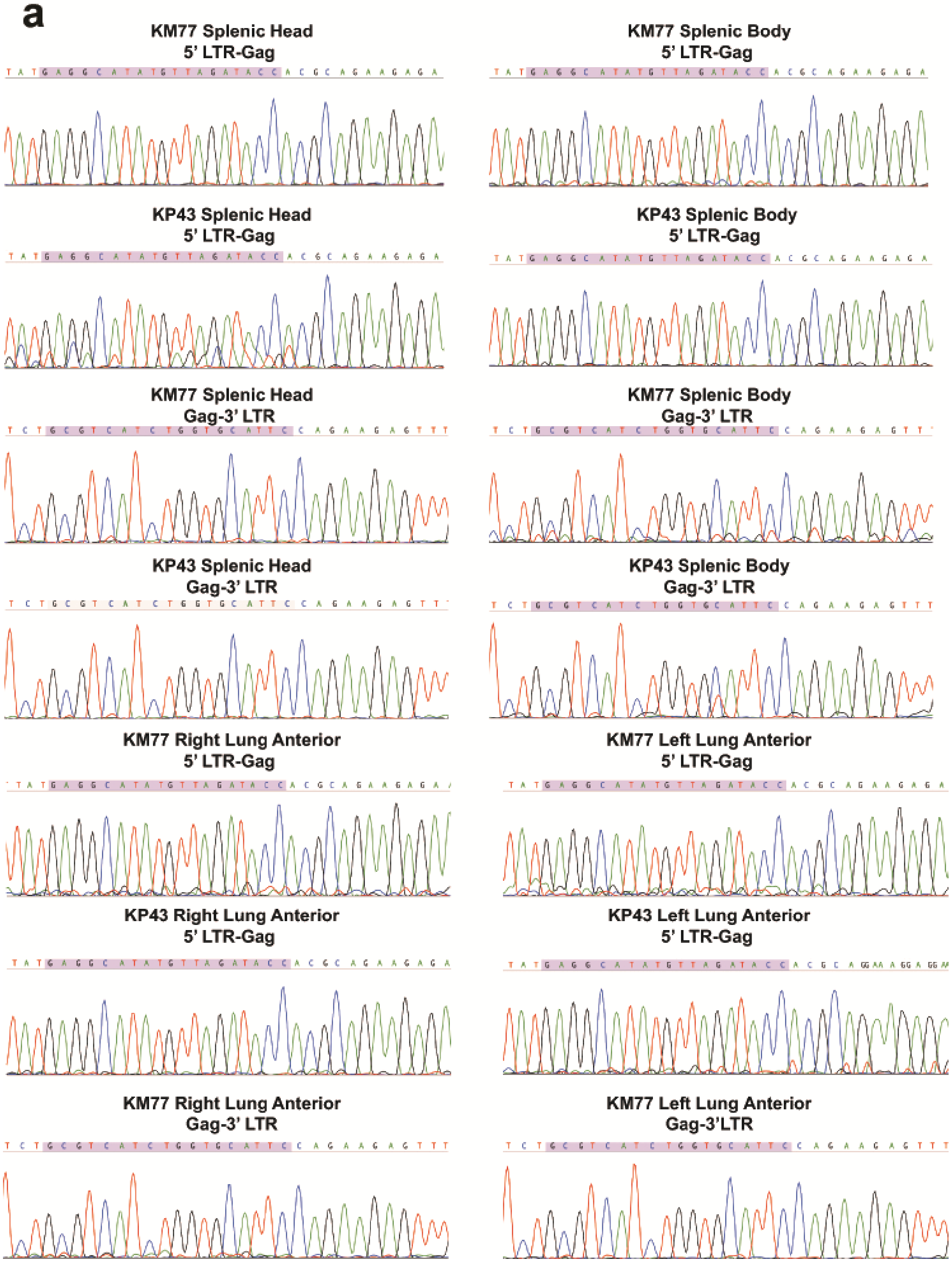
a
KM77 Splenic Head
5' LTR-Gag
KM77 Splenic Body
5' LTR-Gag
KP43 Splenic Head
5' LTR-Gag
KP43 Splenic Body
5' LTR-Gag
KM77 Splenic Head
Gag-3' LTR
KM77 Splenic Body
Gag-3' LTR
KP43 Splenic Head
Gag-3' LTR
KP43 Splenic Body
Gag-3' LTR
KM77 Right Lung Anterior
5' LTR-Gag
KM77 Left Lung Anterior
5' LTR-Gag
KP43 Right Lung Anterior
5' LTR-Gag
KP43 Left Lung Anterior
5' LTR-Gag
KM77 Right Lung Anterior
Gag-3' LTR
KM77 Left Lung Anterior
Gag-3'LTR

b

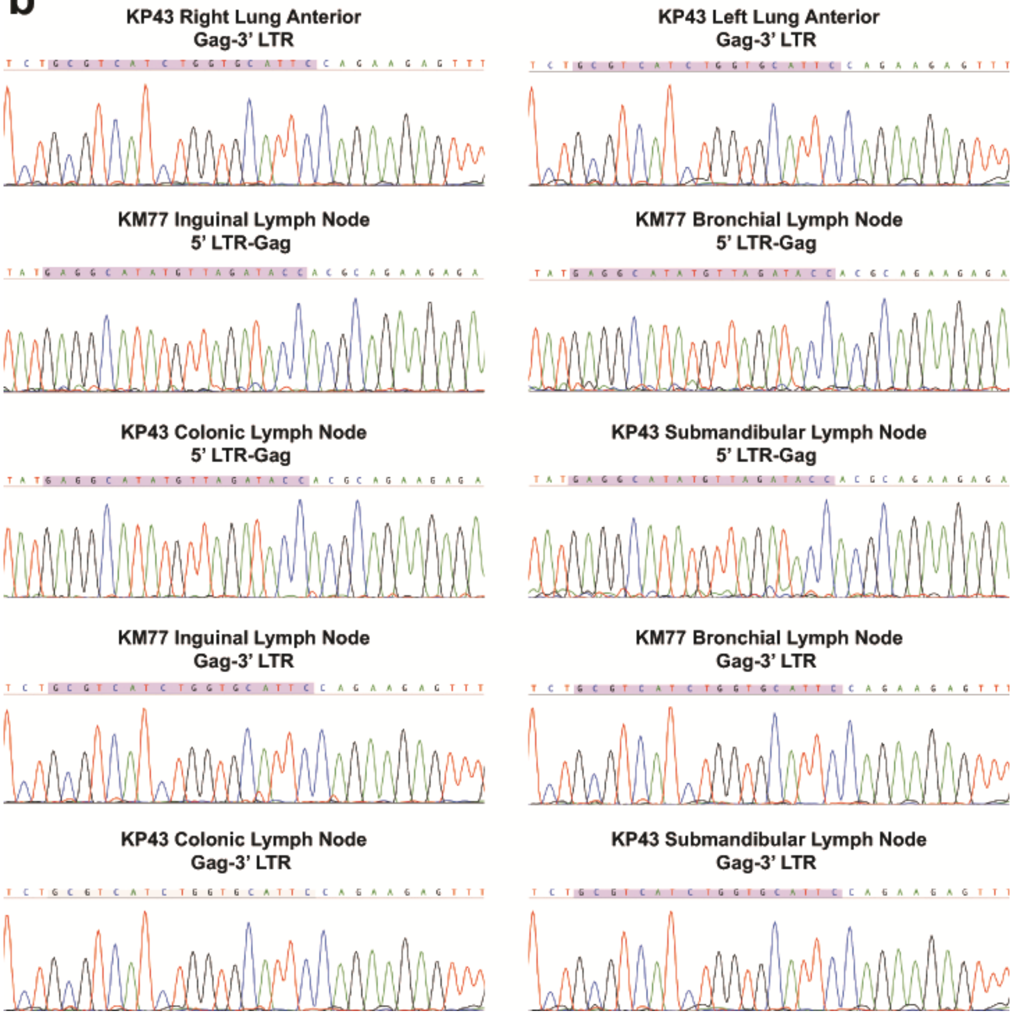
KP43 Right Lung Anterior
Gag-3' LTR
KP43 Left Lung Anterior
Gag-3' LTR
KM77 Inguinal Lymph Node
5' LTR-Gag
KM77 Bronchial Lymph Node
5' LTR-Gag
KP43 Colonic Lymph Node
5' LTR-Gag
KP43 Submandibular Lymph Node
5' LTR-Gag
KM77 Inguinal Lymph Node
Gag-3' LTR
KM77 Bronchial Lymph Node
Gag-3' LTR
KP43 Colonic Lymph Node
Gag-3' LTR
KP43 Submandibular Lymph Node
Gag-3' LTR

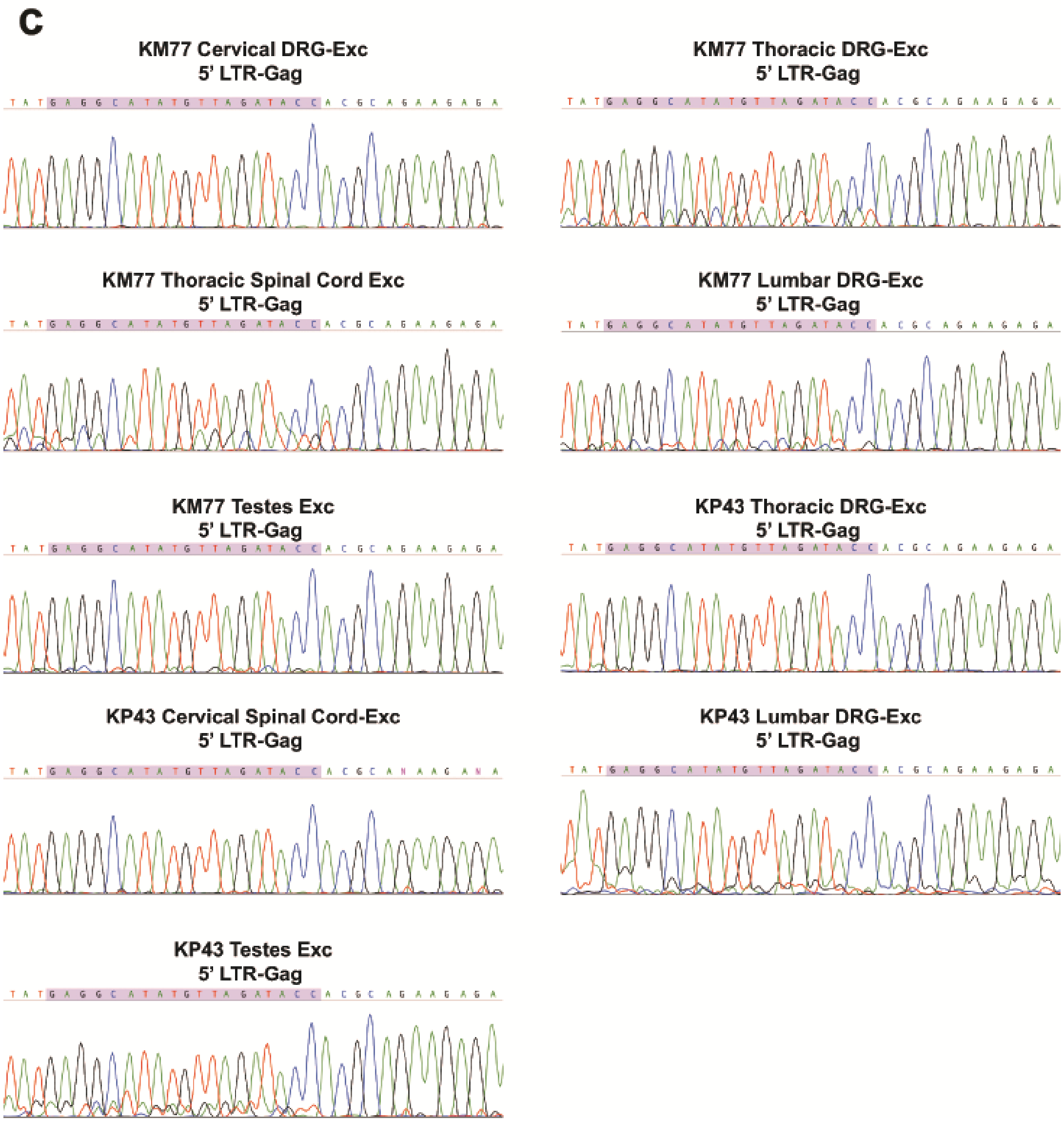
c
KM77 Cervical DRG-Exc
5' LTR-Gag
KM77 Thoracic DRG-Exc
5' LTR-Gag
KM77 Thoracic Spinal Cord Exc
5' LTR-Gag
KM77 Lumbar DRG-Exc
5' LTR-Gag
KM77 Testes Exc
5' LTR-Gag
KP43 Thoracic DRG-Exc
5' LTR-Gag
KP43 Cervical Spinal Cord-Exc
5' LTR-Gag
KP43 Lumbar DRG-Exc
5' LTR-Gag
KP43 Testes Exc
5' LTR-Gag

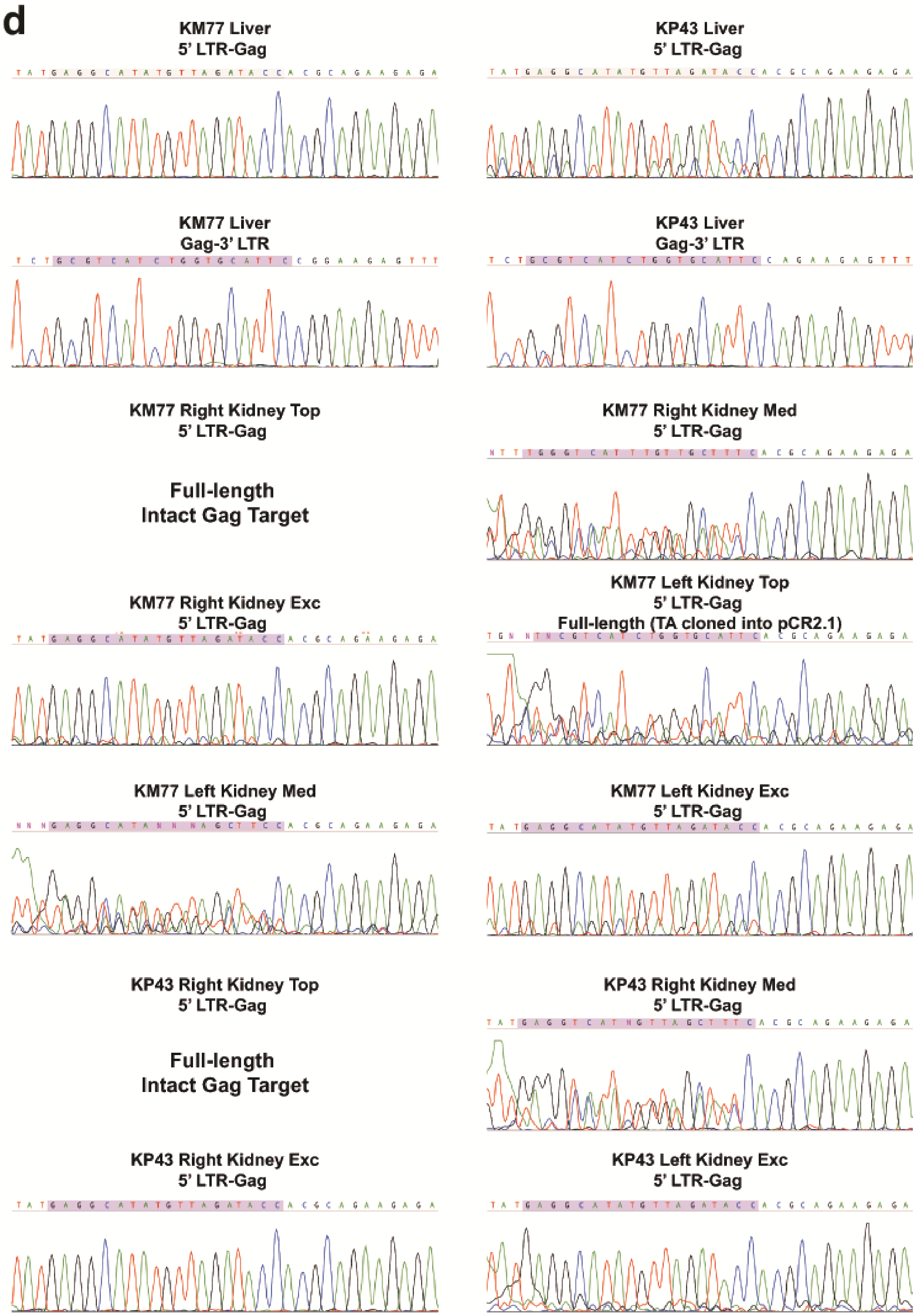
d
KM77 Liver
5' LTR-Gag
KP43 Liver
5' LTR-Gag
KM77 Liver
Gag-3' LTR
KP43 Liver
Gag-3' LTR
KM77 Right Kidney Top
5' LTR-Gag
Full-length
Intact Gag Target
KM77 Right Kidney Med
5' LTR-Gag
KM77 Right Kidney Exc
5' LTR-Gag
KM77 Left Kidney Top
5' LTR-Gag
Full-length (TA cloned into pCR2.1)
KM77 Left Kidney Med
5' LTR-Gag
KM77 Left Kidney Exc
5' LTR-Gag
KP43 Right Kidney Top
5' LTR-Gag
Full-length
Intact Gag Target
KP43 Right Kidney Med
5' LTR-Gag
KP43 Right Kidney Exc
5' LTR-Gag
KP43 Left Kidney Exc
5' LTR-Gag

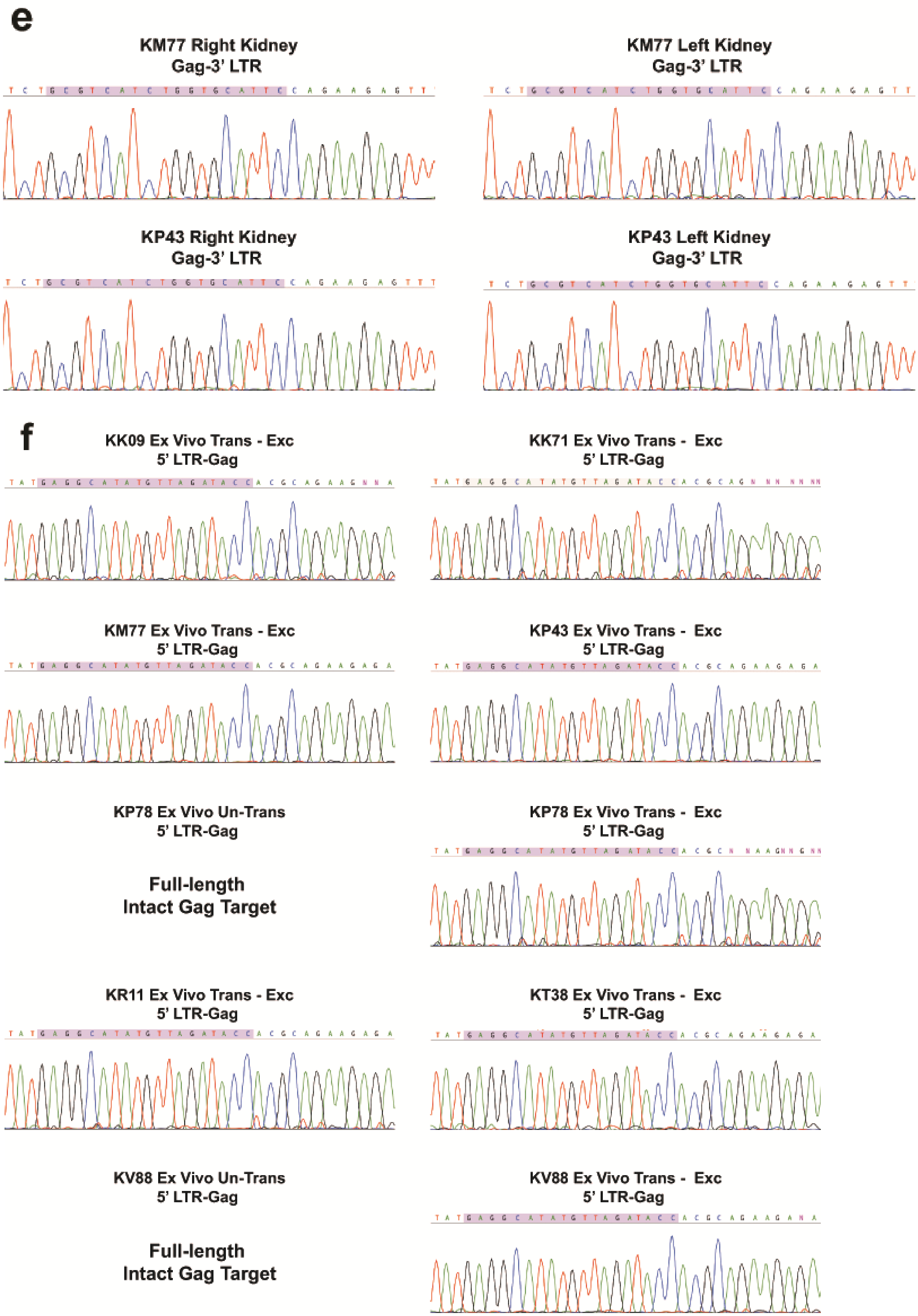
e
KM77 Right Kidney
Gag-3' LTR
KM77 Left Kidney
Gag-3' LTR
KP43 Right Kidney
Gag-3' LTR
KP43 Left Kidney
Gag-3' LTR
f
KK09 Ex Vivo Trans - Exc
5' LTR-Gag
KK71 Ex Vivo Trans - Exc
5' LTR-Gag
KM77 Ex Vivo Trans - Exc
5' LTR-Gag
KP43 Ex Vivo Trans - Exc
5' LTR-Gag
KP78 Ex Vivo Un-Trans
5' LTR-Gag
Full-length
Intact Gag Target
KP78 Ex Vivo Trans - Exc
5' LTR-Gag
KR11 Ex Vivo Trans - Exc
5' LTR-Gag
KT38 Ex Vivo Trans - Exc
5' LTR-Gag
KV88 Ex Vivo Un-Trans
5' LTR-Gag
Full-length
Intact Gag Target
KV88 Ex Vivo Trans - Exc
5' LTR-Gag

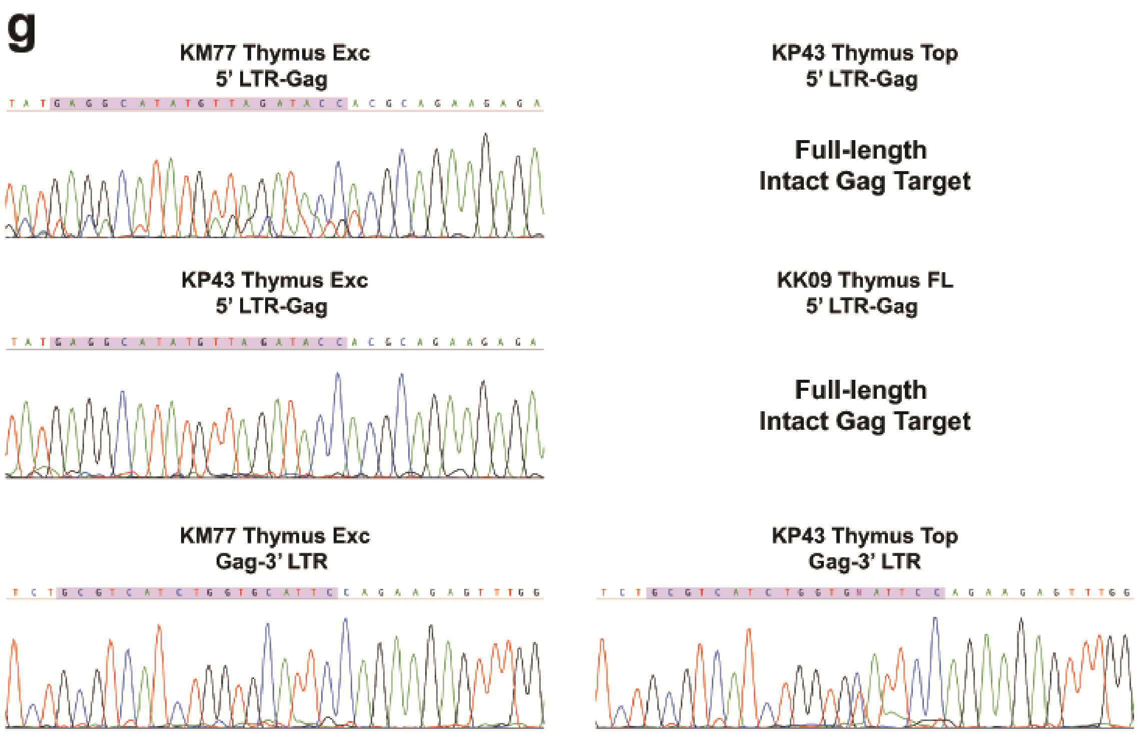

Supplementary Figure 8a-g. Nucleotide sequencing of the 268 and 171 bp amplicons detected after PCR amplification of viral DNA obtained from the various tissues (as denoted) harvested from AAV9-CRISPR SIV-infected animals. Full sequencing data are available in the source file data provided with this paper. Source data are provided as a Source Data file.

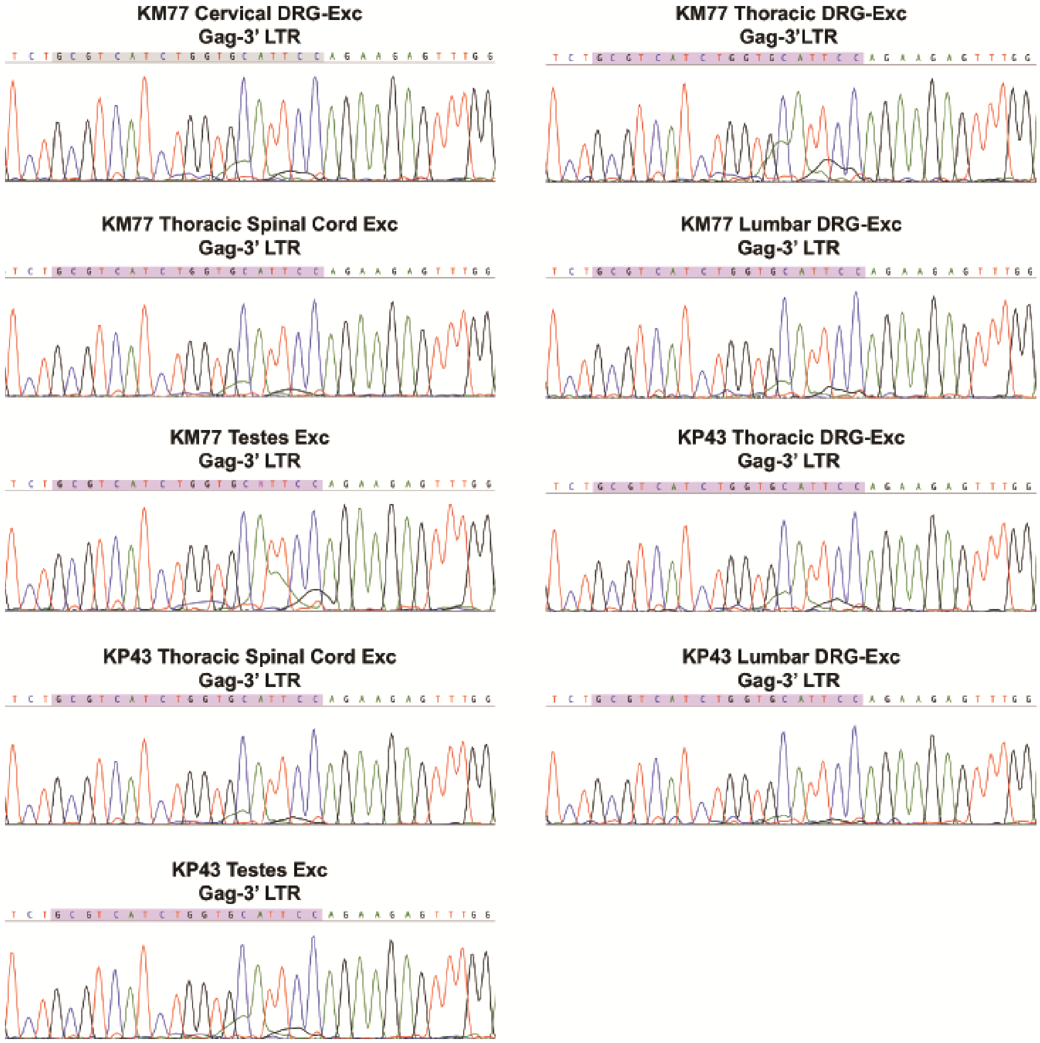

Supplementary Figure 9. Detection of InDel mutations at the sites of breakpoints in several tissues. Full sequencing data are available in the source file data provided with this paper. Source data are provided as a Source Data file.

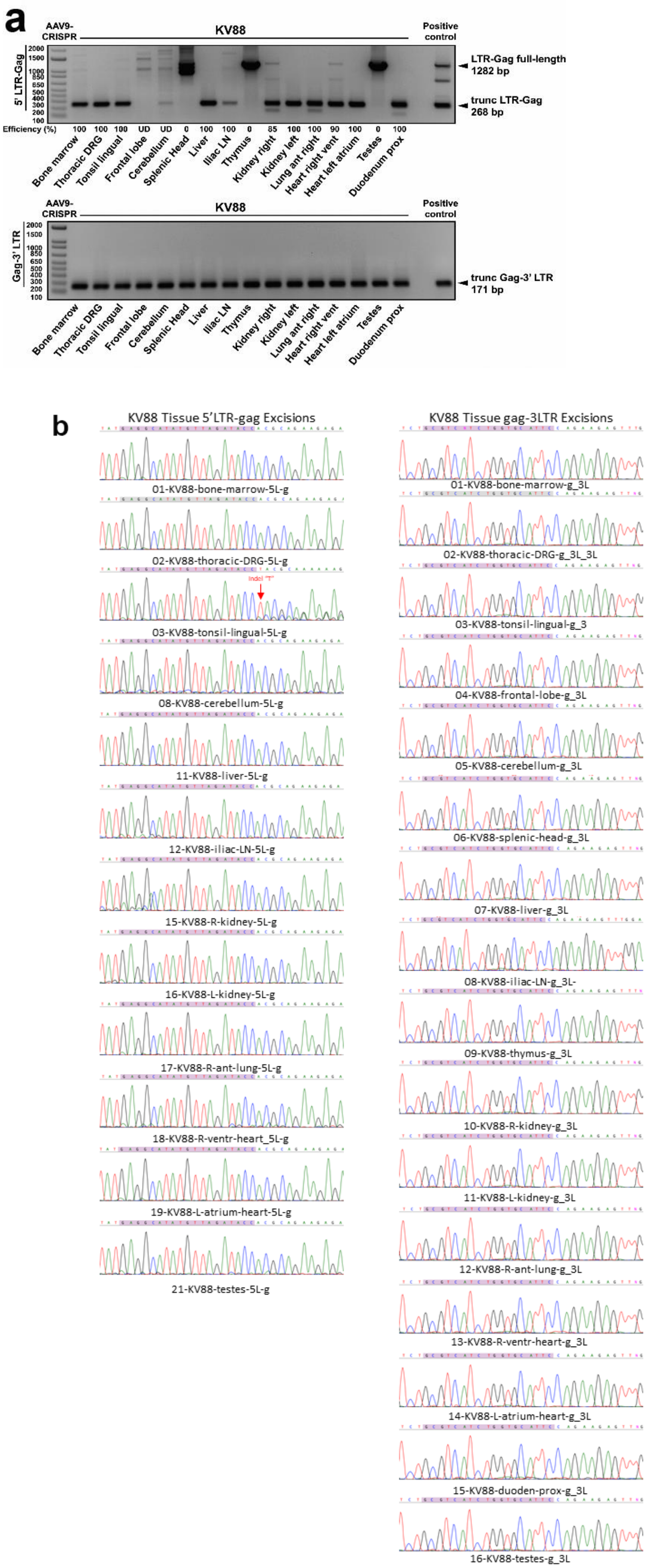
a
AAV9-CRISPR
KV88
Positive control
5' LTR-Gag
2000
1500
1000
850
650
500
400
300
200
100
LTR-Gag full-length 1282 bp
trunc LTR-Gag 268 bp
Efficiency (%)
100 100 100 UD UD 0 100 100 0 85 100 100 90 100 0 100
Bone marrow
Thoracic DRG
Tonsil lingual
Frontal lobe
Cerebellum
Splenic Head
Liver
Iliac LN
Thymus
Kidney right
Kidney left
Lung ant right
Heart right vent
Heart left atrium
Testes
Duodenum prox
AAV9-CRISPR
KV88
Positive control
Gag-3' LTR
trunc Gag-3' LTR 171 bp
Bone marrow
Thoracic DRG
Tonsil lingual
Frontal lobe
Cerebellum
Splenic Head
Liver
Iliac LN
Thymus
Kidney right
Kidney left
Lung ant right
Heart right vent
Heart left atrium
Testes
Duodenum prox
b
KV88 Tissue 5'LTR-gag Excisions
01-KV88-bone-marrow-5L-g
02-KV88-thoracic-DRG-5L-g
Indel "T"
03-KV88-tonsil-lingual-5L-g
08-KV88-cerebellum-5L-g
11-KV88-liver-5L-g
12-KV88-iliac-LN-5L-g
15-KV88-R-kidney-5L-g
16-KV88-L-kidney-5L-g
17-KV88-R-ant-lung-5L-g
18-KV88-R-ventr-heart_5L-g
19-KV88-L-atrium-heart-5L-g
21-KV88-testes-5L-g
KV88 Tissue gag-3LTR Excisions
01-KV88-bone-marrow-g_3L
02-KV88-thoracic-DRG-g_3L_3L
03-KV88-tonsil-lingual-g_3
04-KV88-frontal-lobe-g_3L
05-KV88-cerebellum-g_3L
06-KV88-splenic-head-g_3L
07-KV88-liver-g_3L
08-KV88-iliac-LN-g_3L-
09-KV88-thymus-g_3L
10-KV88-R-kidney-g_3L
11-KV88-L-kidney-g_3L
12-KV88-R-ant-lung-g_3L
13-KV88-R-ventr-heart-g_3L
14-KV88-L-atrium-heart-g_3L
15-KV88-duoden-prox-g_3L
16-KV88-testes-g_3L

Supplementary Figure 10. A. In vivo editing of viral DNA in SIV-infected macaque, KV88, by AAV9-CRISPR. Detection of 268 bp and 171 bp DNA amplicons by PCR in various tissues indicative of the excision of viral proviral DNA positioned between 5' LTR and Gag, and Gag-3'LTR, respectively after treatment with AAV9-CRISPR. The percent excision efficiency in vivo (Efficiency (%)) shown under the PCR was calculated in 5' LTR-Gag by quantification of the excised band (Trunc.) divided by the full-length band (FL) times 100 percent. UD=undetermined. **B.** DNA sequencing data illustrating the remaining viral DNA fragments shown in Panel A after the excision of intervening DNA between the 5' LTR and Gag and Gag to 3' LTR. The position of DNA breakpoints and InDel mutations after editing are highlighted. Full sequencing data are available in the source file data provided with this paper. Source data are provided as a Source Data file.

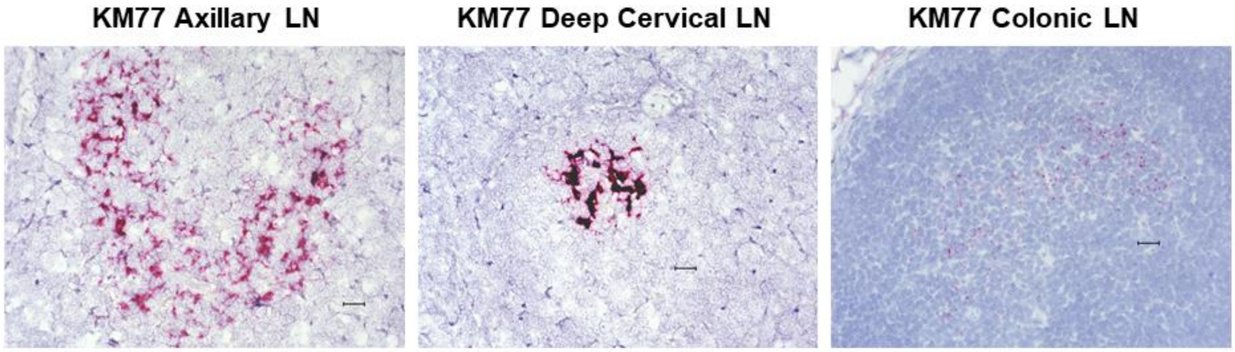

Supplementary Figure 11. Detection of Cas9 DNA in axillary lymph node, deep cervical lymph node and colonic lymph node tissues. Cas9 DNA (purple) was detected using DNAScope in situ in axillary lymph node, deep cervical lymph node and colonic lymph node of animal KM77, who received in vivo AAV9 CRISPR-Cas9. No Cas9 DNA was found in any lymph nodes of the control animal KK09. Scale bar= 20μm. Representative images were repeated independently with similar results. Source data are provided as a Source Data file.

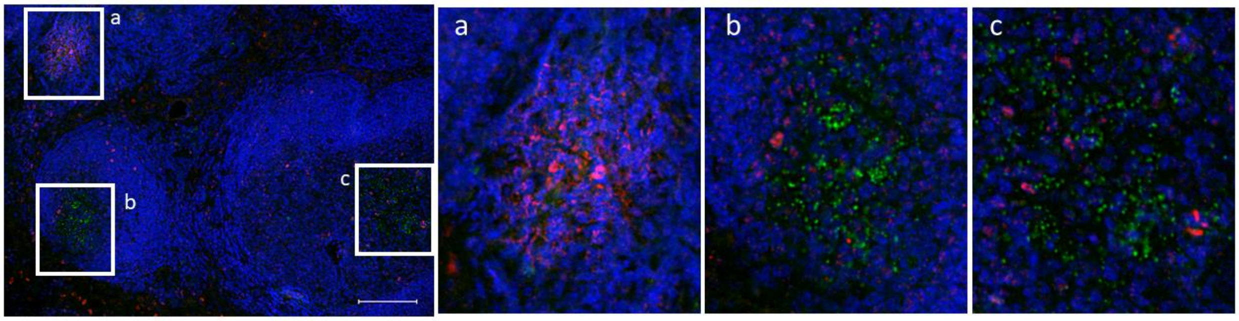

Supplementary Figure 12. Cas9 RNA and SIV RNA were detected using dual RNAScope technology in SIV infected macaque KV88, who received in vivo CRISPR. SIV RNA is in green and Cas9 RNA is in red. The left picture is a 20X image of a mesenteric LN. The white boxes a, b, and c are enlarged images of sections of the 20X image as shown. These images show a lack of co-expression of SIV RNA and Cas9 RNA. These images show a loss/lack of SIV signal (green) in locations where the AAV9 Cas9 (red) is located. Scale bar= 100μm. Representative images were repeated independently with similar results. Source data are provided as a Source Data file.

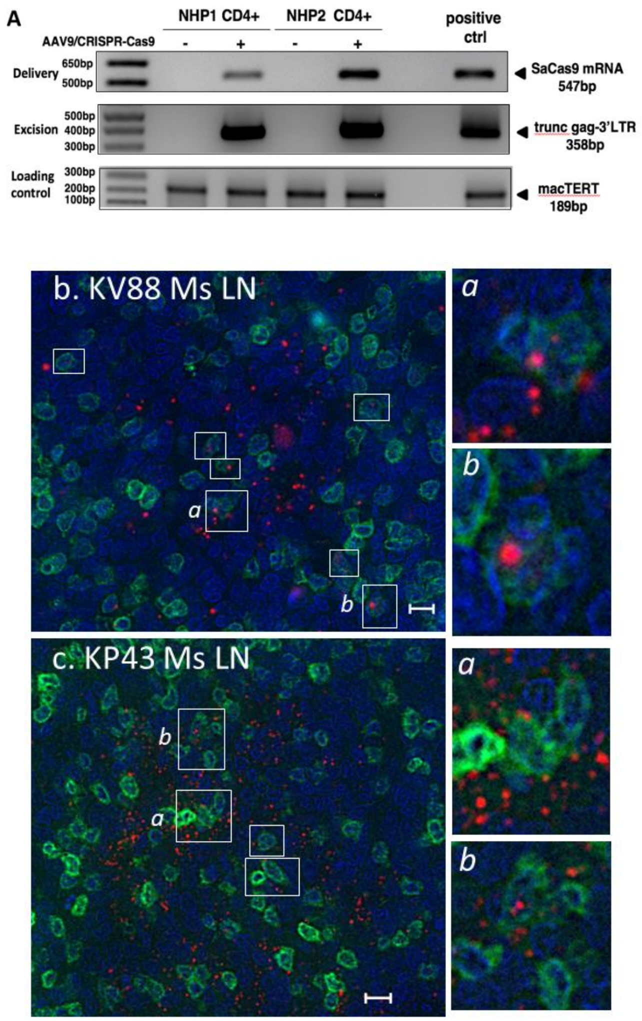

Supplementary Figure 13. AAV9 CRISPR-Cas9 can be delivered and excise SIV viral DNA in CD4+ T cells. a. CD4+ T cells were isolated from fresh blood from two SIV infected ART-treated animals (NHP1 and NHP2, from an ongoing study) and AAV/CRISPR-Cas9 was delivered ex vivo. The presence of Cas9 mRNA was confirmed in the transduced animals SaCas9 mRNA (547 bp) as well as viral excision of the Gag-3' LTR as seen by the amplicon at 358 bp. Amplicons were sequence verified by Sanger sequencing. Macaque TERT was used as a loading control (mac TERT 189 bp). AAV9 delivered CRISPR-Cas9 and viral excision occurred in CD4+ T cells ex vivo. **b-c.** Cas9 DNAScope (red) followed CD3 immunohistochemistry (green) was performed in mesenteric lymph nodes (MS LN) of KV88 (b) and KP43 (c) animals treated with CRISPR (40X image). Insert panels a and b for each show enlargements of CD3+ cells that are Cas9 DNA

positive. White boxes are identifying CD3+ T cells that are also Cas9 DNA+. Scale bar= 10μm. Representative images were repeated independently with similar results. Source data are provided as a Source Data file.
