# Supplementary material for: CRISPR based editing of SIV proviral DNA in ART treated non-human primates
Source: Nat Commun. 2020 Nov 27;11:6065. doi: 10.1038/s41467-020-19821-7 (PMC7695718; doi:10.1038/s41467-020-19821-7)
Supplement: Supplementary file 2 — Reporting Summary [file 41467_2020_19821_MOESM2_ESM.pdf]

nature research

Corresponding author(s): Kamel Khalili, Ph.D., Tricia H. Burdo, Ph.D., Andrew MacLean, Ph.D, Binhua Ling, M.D., Ph.D.

Last updated by author(s): Sep 29, 2020

Reporting Summary

Nature Research wishes to improve the reproducibility of the work that we publish. This form provides structure for consistency and transparency in reporting. For further information on Nature Research policies, see Authors & Referees and the Editorial Policy Checklist .

Statistics

For all statistical analyses, confirm that the following items are present in the figure legend, table legend, main text, or Methods section.

n/a	Confirmed	
☐	☒	The exact sample size (*n*) for each experimental group/condition, given as a discrete number and unit of measurement
☐	☒	A statement on whether measurements were taken from distinct samples or whether the same sample was measured repeatedly
☒	☐	The statistical test(s) used AND whether they are one- or two-sided<br>*Only common tests should be described solely by name; describe more complex techniques in the Methods section.*
☒	☐	A description of all covariates tested
☒	☐	A description of any assumptions or corrections, such as tests of normality and adjustment for multiple comparisons
☒	☐	A full description of the statistical parameters including central tendency (e.g. means) or other basic estimates (e.g. regression coefficient) AND variation (e.g. standard deviation) or associated estimates of uncertainty (e.g. confidence intervals)
☒	☐	For null hypothesis testing, the test statistic (e.g. *F*, *t*, *r*) with confidence intervals, effect sizes, degrees of freedom and *P* value noted<br>*Give P values as exact values whenever suitable.*
☒	☐	For Bayesian analysis, information on the choice of priors and Markov chain Monte Carlo settings
☒	☐	For hierarchical and complex designs, identification of the appropriate level for tests and full reporting of outcomes
☒	☐	Estimates of effect sizes (e.g. Cohen's *d*, Pearson's *r*), indicating how they were calculated

Our web collection on statistics for biologists contains articles on many of the points above.

Software and code

Policy information about availability of computer code

Data collection	For flow cytometry the data were captured using BD LSRFortessa Flow Cytometer.<br>Plasma viral data was obtained using 7900HT fast Thermo Fisher following the protocol in Monjure, et al., Optimization of PCR for quantification of simian immunodeficiency virus genomic RNA in plasma of rhesus macaques (Macaca mulatta) using armored RNA. Journal of Medical Primatology, 43: 31–43.<br>CBCs were collecyed using Sysmex XN-1000V and Chemistries were collected using Beckman Coulter AU480.<br>ddPCR data were acquired using QX200 droplet reader (Bio-Rad Laboratories, Hercules, CA, USA)
Data analysis	For flow cytometry the data were analyzed using FlowJo Version 10.5.3.<br>All qPCR DNA/RNA samples were analyzed by Graph Pad Prism 7.0 software (La Jolla, CA).<br>ddPCR data were analyzed using QuantaSoft software (Bio-Rad Laboratories, Hercules, CA, USA)

For manuscripts utilizing custom algorithms or software that are central to the research but not yet described in published literature, software must be made available to editors/reviewers. We strongly encourage code deposition in a community repository (e.g. GitHub). See the Nature Research guidelines for submitting code & software for further information.

Data

Policy information about availability of data

All manuscripts must include a data availability statement. This statement should provide the following information, where applicable:

- Accession codes, unique identifiers, or web links for publicly available datasets
- A list of figures that have associated raw data
- A description of any restrictions on data availability

Data statement has been added to the methods section of the manuscript and the figure legends. All data are available on request from the authors. All data generated or analyzed during the study are included in this published article (and its supplementary files).

Field-specific reporting

Please select the one below that is the best fit for your research. If you are not sure, read the appropriate sections before making your selection.

- [x] Life sciences
- [ ] Behavioural & social sciences
- [ ] Ecological, evolutionary & environmental sciences

For a reference copy of the document with all sections, see nature.com/documents/nr-reporting-summary-flat.pdf

Life sciences study design

All studies must disclose on these points even when the disclosure is negative.

Sample size	No statistical method was used to predetermine the sample size. This was a proof of concept study.
Data exclusions	No data were excluded.
Replication	Two animals were given CRISPR/Cas9 gene editing at the same time and later the 3rd was given an in vivo CRISPR/Cas9 for reproducibility. All real-time PCR assays, PCR amplification and ddPCR were repeated multiple times by different investigators. A reproducibility section has been added to the methods section.
Randomization	The 3 animals that were given CRISPR/Cas9 were picked randomly as well as the one that was sacrificed as a control. All animals chosen were AAV9 neutralizing antibody negative before the initiation of the study.
Blinding	The investigators were blinded during the analysis for qPCR, ddPCR, DNAScope, excision assays.

Reporting for specific materials, systems and methods

We require information from authors about some types of materials, experimental systems and methods used in many studies. Here, indicate whether each material, system or method listed is relevant to your study. If you are not sure if a list item applies to your research, read the appropriate section before selecting a response.

Materials & experimental systems

n/a	Involved in the study	
[ ]	[x]	Antibodies
[ ]	[x]	Eukaryotic cell lines
[x]	[ ]	Palaeontology
[ ]	[x]	Animals and other organisms
[x]	[ ]	Human research participants
[x]	[ ]	Clinical data

Methods

n/a	Involved in the study	
[x]	[ ]	ChIP-seq
[ ]	[x]	Flow cytometry
[x]	[ ]	MRI-based neuroimaging

Antibodies

Antibodies used	Aqua Live/Dead AmCyan (Invitrogen, Thermo Fisher)<br>CD3: clone SP34-2, APC-Cy7 (BD Biosciences)<br>CD4: clone OKT4, BV650 (Biolegend)<br>CD8: clone SK1, AL488 (Biolegend)<br>CD3: Cat# A0452; DAKO
Validation	Antibodies were tested and titrated using Quality Protocols by members of the TNPRC flow cytometry core. All antibodies were verified for cross reactivity in rhesus macaques through the NIH-funded Non-human Primate Reagent Resource.

Eukaryotic cell lines

Policy information about cell lines

Cell line source(s)	HEK293 T cells, ATCC
Authentication	Per ATCC as described in the data sheet. All cell lines were directly received from ATCC, who routinely verifies all cell lines.
Mycoplasma contamination	Routinuely tested for mycoplasma in house MD Bioscience's PCR mycoplasma detection kit. All cell lines tested negative for mycoplasma.

Commonly misidentified lines
(See ICLAC register)

none used

Animals and other organisms

Policy information about studies involving animals; ARRIVE guidelines recommended for reporting animal research

Laboratory animals	Maccaca mulatta, Chinese origin, male, ages 5 years old (5.18, 5.22, 5.34 & 6.01 years).
Wild animals	No wild animals were used in this study.
Field-collected samples	No field collected samples were used in this study
Ethics oversight	Animals were housed at the Tulane National Primate Research Center (TNPRC; Covington, LA). All animals used in this study were handled in strict accordance with American Association for Accreditation of Laboratory Animal Care with the approval of the Institutional Animal Care and Use Committee of Tulane University.

Note that full information on the approval of the study protocol must also be provided in the manuscript.

Flow Cytometry

Plots

Confirm that:

- [x] The axis labels state the marker and fluorochrome used (e.g. CD4-FITC).
- [x] The axis scales are clearly visible. Include numbers along axes only for bottom left plot of group (a 'group' is an analysis of identical markers).
- [x] All plots are contour plots with outliers or pseudocolor plots.
- [x] A numerical value for number of cells or percentage (with statistics) is provided.

Methodology

Sample preparation	PBMCs were first stained with live/dead stain, washed with PBS containing 2% fetal calf serum, and then stained with anti-CD3 APC-Cy7 (SP34-2, BD Biosciencs), anti-CD4 BV450 (OKT4, Biolegend), and anti-CD8 AL488 (SK1, Biolegend) monoclonal antibodies, and incubated for 30 min at room temperature in the dark. Fixed cells were captured using BD LSRFortessa flow cytometer, at least 20,000 events were collected by gating on cell singlets, live cells, CD3+ lymphocytes, then further gating CD4+ and CD8+ lymphocytes.
Instrument	For flow cytometry the data were captured using BD LSRFortessa Flow Cytometer.
Software	For flow cytometry the data were analyzed using FlowJo Version 10.5.3.
Cell population abundance	No sorting was performed so no post-sorting purification was performed.
Gating strategy	At least 20,000 events were collected by gating on cell singlets, live cells, CD3+ lymphocytes, then further gating CD4+ and CD8+ lymphocytes.

- [x] Tick this box to confirm that a figure exemplifying the gating strategy is provided in the Supplementary Information.

October 2018
